# Compartmentalization of Melanin Biosynthetic Enzymes Contributes to Self-Defense against Intermediate Compound Scytalone in *Botrytis cinerea*

**DOI:** 10.1128/mBio.00007-21

**Published:** 2021-03-23

**Authors:** Xue Chen, Chuanxi Zhu, Yantao Na, Dandan Ren, Chenghua Zhang, Yifan He, Yiwen Wang, Sheng Xiang, Weiheng Ren, Yina Jiang, Ling Xu, Pinkuan Zhu

**Affiliations:** a School of Life Sciences, East China Normal University, Shanghai, China; Cornell University

**Keywords:** *Botrytis cinerea*, DHN melanin, scytalone, subcellular trafficking, endosome, peroxisome

## Abstract

In filamentous fungi, 1,8-dihydroxynaphthalene (DHN) melanin is a major component of the extracellular matrix, endowing fungi with environmental tolerance and some pathogenic species with pathogenicity. However, the subcellular location of the melanin biosynthesis pathway components remains obscure. Using the gray mold pathogen Botrytis cinerea, the DHN melanin intermediate scytalone was characterized via phenotypic and chemical analysis of mutants, and the key enzymes participating in melanin synthesis were fused with fluorescent proteins to observe their subcellular localizations. The Δ*bcscd1* mutant accumulated scytalone in the culture filtrate rather than in mycelium. Excessive scytalone appears to be self-inhibitory to the fungus, leading to repressed sclerotial germination and sporulation in the Δ*bcscd1* mutant. The BcBRN1/2 enzymes responsible for synthesizing scytalone were localized in endosomes and found to be trafficked to the cell surface, accompanied by the accumulation of BcSCD1 proteins in the cell wall. In contrast, the early-stage melanin synthesis enzymes BcPKS12/13 and BcYGH1 were localized in peroxisomes. Taken together, the results of this study revealed the subcellular distribution of melanin biosynthetic enzymes in B. cinerea, indicating that the encapsulation and externalization of the melanin synthetic enzymes need to be delicately orchestrated to ensure enzymatic efficiency and protect itself from the adverse effect of the toxic intermediate metabolite.

## Abstract (precis)

The devastating gray mold pathogen *Botrytis cinerea* propagates via melanized conidia and sclerotia. This study reveals that the sclerotial germination of *B. cinerea* is differentially affected by different enzymes in the melanin synthesis pathway.

## INTRODUCTION

Botrytis cinerea is a typical necrotrophic plant pathogen with a wide host range that causes severe economic losses to pre- and postharvest crops and, thus, is one of the most notorious cosmopolitan phytopathogenic fungi (1). The control of this pathogen is very difficult due to the rapid evolution of resistance against the main classes of fungicides (2, 3), diversified virulence strategies to actively promote plant susceptibility, and high survivability under unfavorable environmental conditions (4–6). Moreover, absolute resistance to B. cinerea is rarely found in host cultivars (7, 8). Consequently, research focusing on the life cycle and pathogenicity mechanisms of *B. cinerea* is essential for developing new disease management strategies.

During the growth season of host crops, *B. cinerea* can infect plant tissues and consume plant nutrients to develop into mycelial biomass and propagating conidia, which are dispersed for repeated infection cycles (9). Under unfavorable conditions, such as the winter season, the fungus prefers to lurk in the host tissue or debris materials in the form of sclerotia to resist environmental adversities (10). When suitable conditions appear in the next spring, the sclerotia germinate and produce hyphae and asexual conidia or cross with microconidia of the opposite mating type to develop into sexual ascospores, thus initiating the infection cycles (9, 10). Interestingly, both conidia and sclerotia of *B. cinerea* are characteristically gray to black pigmented as a result of melanin accumulation.

Melanin is a macromolecular substance formed by polymerizing phenols or indole monomers and complexing with saccharides or proteins. It is widely found in animals, plants, and microorganisms (11). In fungi, various types of melanin have been identified, among which l-3,4-dihydroxyphenylalanine (l-DOPA) and 1,8-dihydroxynaphthalene (1,8-DHN) melanin have been found in basidiomycetous and ascomycetous species, respectively (12, 13). Fungal melanin can be formed by enzymatic reaction or auto-oxidative polymerization in the medium, and granular aggregation of this polymer can be observed on cell walls (14, 15).

Melanin mainly plays two roles in fungi: one is protecting fungi from various adversities such as drying, extreme temperatures, free radicals, UV irradiation, ionizing radiation, osmotic stress, and fungicides (12, 14, 16), and the other is acting as a virulence factor in certain pathogenic fungi (17–19). As for some phytopathogens, e.g., Magnaporthe oryzae and *Colletotrichum* spp., melanization is a prerequisite for the infection structure appressorium to accumulate very high turgor pressure to penetrate the host epidermis (20–23). In some human-pathogenic fungi such as Cryptococcus neoformans (24, 25) and Aspergillus fumigatus (26, 27), melanin in the cell wall protects from the attack of the host immune system, thus ensuring the virulence of these pathogens. Moreover, a human host C-type lectin receptor was found to recognize fungal DHN melanin as an immunologically active component commonly found on fungi, indicating an essential role in protective antifungal immunity in both mice and humans (28). Therefore, fungal melanin could also be perceived as a molecular pattern of pathogens by their hosts.

In *B. cinerea*, it has been shown that 1,8-DHN melanin is synthesized via a polyketide synthase (PKS) pathway (see Fig. S1 in the supplemental material). With acetyl coenzyme A as a substrate, T4HN is synthesized by the polyketide synthase BcPKS12, or BcPKS13 together with BcYGH1, and T4HN is then reduced to scytalone by the action of the 1,3,6,8-tetrahydroxynaphthalene (T4HN) reductase BcBRN1 or BcBRN2. Scytalone is dehydrated into 1,3,8-trihydroxynaphthalene (T3HN) by scytalone dehydratase (BcSCD1), and T3HN is reduced by BcBRN1/2 to form vermelone, which is dehydrated by BcSCD1 to form 1,8-DHN (29). Although melanin seems to not be required for the virulence of *B. cinerea*, the fungus accumulates melanin in conidia and sclerotia, suggesting that DHN melanin should play roles in the formation of asexual reproductive structures. Additionally, it has been found that a single-nucleotide deletion in the transcription factor gene *Bcsmr1* caused sclerotial-melanogenesis deficiency in *B. cinerea* (30), and the melanin-deficient sclerotia were more susceptible to mycoparasites than the wild type (WT) (31). However, the cytological regulation mechanisms of melanin biosynthesis and subsequent cell wall deposition of melanin remain to be clarified in *B. cinerea*.

10.1128/mBio.00007-21.1 FIG S1 DHN melanin synthesis pathway in *B. cinerea* (J. Schumacher, Mol Microbiol, 99:729–748, 2016, https://doi.org/10.1111/mmi.13262). Abbreviations: AT4HN, 2-acetyl-1,3,6,8-tetrahydroxynaphthalene; T4HN, 1,3,6,8-tetrahydroxynaphthalene; scytalone, 3,6,8-trihydroxy-3,4-dihydronaphthalene-1(2-hydrogen); T3HN, 1,3,8-trihydroxynaphthalene; vermelone, 3,4-dihydro-3,8-dihydroxy-1(2H)-naphthalenone; 1,8 DHN, 1,8-dihydroxynaphthalene; BcPKS12/13, polyketide synthases; BcYGH1, hydrolases; BcBRN1/2, T4HN reductases; BcSCD1, scytalone dehydratase; tricyclazole, inhibitor of T4HN reductases. Download FIG S1, TIF file, 0.09 MB. Copyright © 2021 Chen et al. 2021 Chen et al. https://creativecommons.org/licenses/by/4.0/ This content is distributed under the terms of the Creative Commons Attribution 4.0 International license.

In this study, we first performed phenotypic analysis of the nonmelanized sclerotium mutants Δ*bcpks12* and Δ*bcscd1*. The results showed that only the Δ*bcscd1* mutant was deficient in sclerotium germination with sporogenesis, leading to the hypothesis of a side effect of DHN melanin intermediate accumulation. We further found that the Δ*bcscd1* mutant accumulated scytalone in the culture filtrate rather than the mycelium, and excessive scytalone appears to be self-inhibitory to the fungus. Furthermore, we report that there is compartmentalization of the melanin synthesis enzymes. These findings revealed how the fungus orchestrates melanin biosynthesis and protects itself from the toxic effect of the intermediate.

## RESULTS

### Sclerotial germination of *B. cinerea* is differentially affected by the loss of different melanin synthesis genes.

Sclerotia are important for *B. cinerea* to fulfill its complete life cycle, and one characteristic of these structures is that they are highly pigmented with melanin. To investigate the role of melanin synthesis in sclerotial performances, the Δ*bcpks12* and Δ*bcscd1* mutants, both of which produce melanin-deficient sclerotia (29), were analyzed in this study.

**FIG 1:**
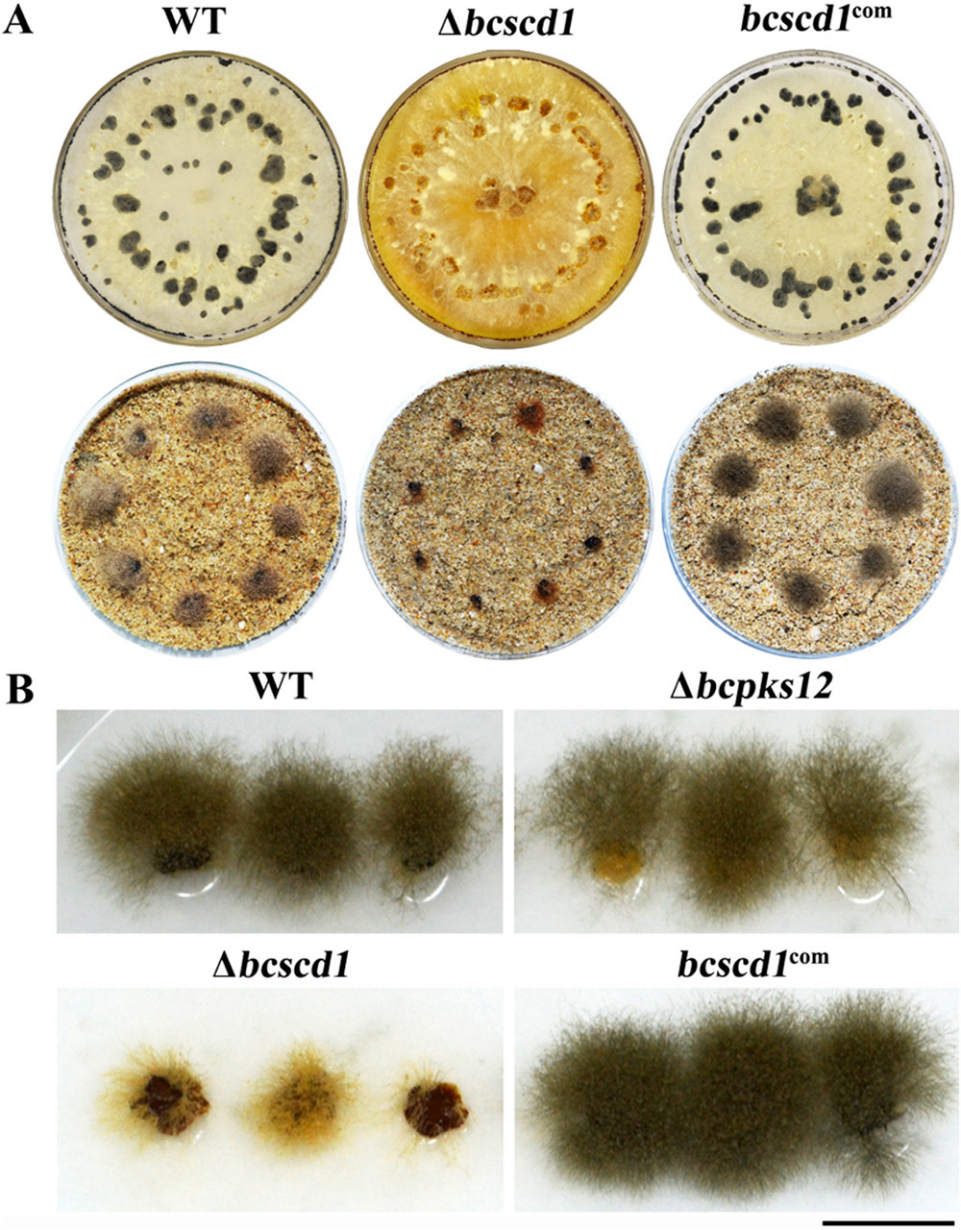
(A) Sclerotia formed by the WT, Δ*bcscd1*, and *bcscd1*^com^ strains (top row) and their germination in wetted sand soil after incubation for 1 month (bottom row). (B) Sclerotial germination is attenuated in the Δ*bcscd1* strain but not in the Δ*bcpks12* strain. Bar, 1 cm.

As one of the basic missions for sclerotia is to generate mycelium and conidia as initial infection sources in the new growth season after dormancy, we analyzed the germination of wild-type and mutant sclerotia and found remarkable differences between the tested strains in sclerotial germination and conidial production by the mycelia developed from the sclerotia (Fig. 1). Of note, germination and subsequent hyphal development of Δ*bcscd1* sclerotia were significantly suppressed, in contrast to the wild-type and the Δ*bcpks12* mutant sclerotia. Consequently, sporulation from the germinating sclerotia was also substantially reduced in the Δ*bcscd1* mutant (Fig. 1B).

The sclerotial germination defects of the Δ*bcscd1* mutant were completely restored by reintroducing the wild-type copy of *Bcscd1* into the mutant (Fig. 1), suggesting that the loss of *Bcscd1* is indeed responsible for the attenuated germination of sclerotia. Although sclerotia of both the Δ*bcpks12* and Δ*bcscd1* mutants are deficient in melanization, the Δ*bcpks12* sclerotia showed normal germination as for the wild type. Notably, the Δ*bcscd1* mutant produced orange mycelium, showing reduced sporulation both in culture and in plants (see Fig. S2 in the supplemental material). These data led to the hypothesis that sclerotial germination with sporogenesis of *B. cinerea* is affected not by the absence of melanin but by the interruption of certain key enzymes of the melanin synthesis pathway.

10.1128/mBio.00007-21.2 FIG S2 Morphological phenotypes expressed by the wild type and mutants of *B. cinerea*. (A) Colony development and sclerotium formation on CM cultured under constant light or dark. (B) Sclerotium production after 10 days of incubation in the dark. (C) Spore production after 10 days of incubation under continuous illumination. (D) Spore production on tomato leaves (after 5 days of incubation). Download FIG S2, TIF file, 2.1 MB. Copyright © 2021 Chen et al. 2021 Chen et al. https://creativecommons.org/licenses/by/4.0/ This content is distributed under the terms of the Creative Commons Attribution 4.0 International license.

### Accumulation of scytalone in the Δ*bcscd1* mutant impairs sclerotium germination and sporulation.

**FIG 2:**
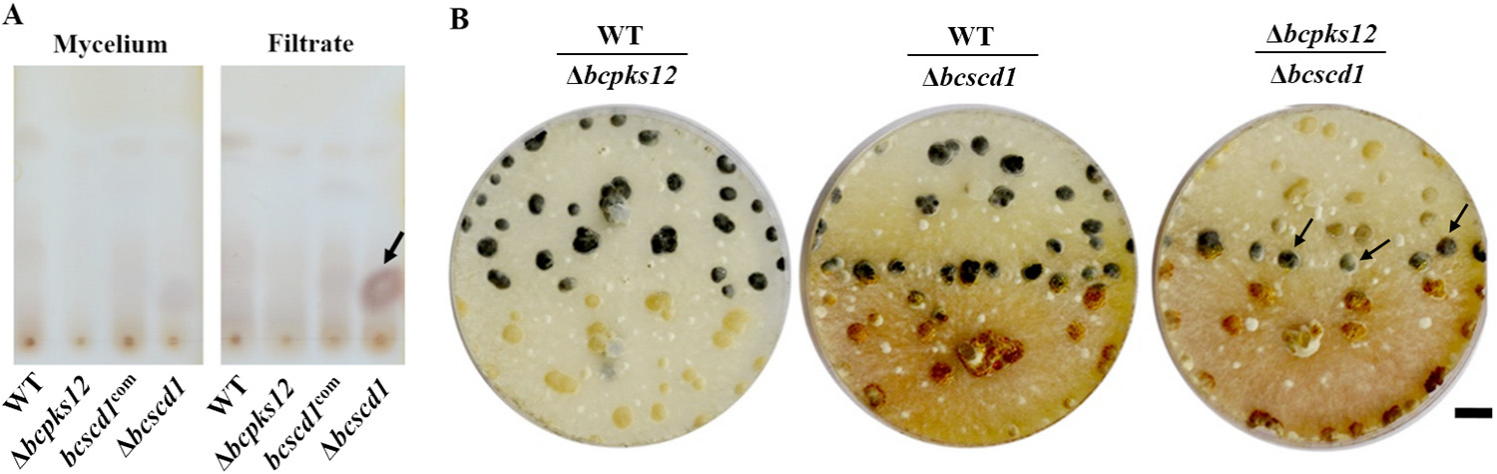
A melanin intermediate is deposited by the Δ*bcscd1* mutant extracellularly. (A) Thin-layer chromatography (TLC) analysis of crude extracts of hyphal homogenates and culture filtrates of the wild type (WT) and three mutant strains of *B. cinerea*. The arrow indicates the strong signal detected in the culture filtrate of the Δ*bcscd1* mutant. (B) The Δ*bcpks12* sclerotia gradually regained melanization when they were cocultured with the Δ*bcscd1* mutant, suggesting that the compound secreted by the Δ*bcscd1* mutant was utilized by the Δ*bcpks12* mutant to remedy its defect in melanin synthesis in sclerotia. Bar, 1 cm.

To define the defect in sclerotium germination of the Δ*bcscd1* mutant, we attempted to extract the intermediate from both hyphal and culture filtrate fractions of this fungus. First, crude extracts from 2-week-old cultures of the wild type and three mutants (Δ*bcpks12*, Δ*bcscd1*, and *bcscd1*^com^) were analyzed by thin-layer chromatography (TLC). No obvious metabolite was detected in the mycelial fraction of all test strains; however, a strong signal was detected only in the culture filtrate fraction of the Δ*bcscd1* mutant (Fig. 2A). This compound was further purified by column chromatography and identified as 3,6,8-trihydroxy-3,4-dihydronaphthalene-1(2-hydrogen) phenol (scytalone) using nuclear magnetic resonance (NMR) and mass spectroscopic analyses (Fig. S3 and S4). Coculturing of Δ*bcpks12* and Δ*bcscd1* mutant strains led to sclerotial melanization of the Δ*bcpks12* but not the Δ*bcscd1* strain, suggesting that the scytalone accumulated by the Δ*bcscd1* strain can be utilized by the Δ*bcpks12* strain to restore its melanin synthesis in sclerotia (Fig. 2B). These results strongly suggested that the melanin intermediate scytalone was secreted extracellularly and accumulated in the Δ*bcscd1* cultures. Furthermore, it is validated that BcSCD1 functions downstream of BcPKS12 in the melanin synthetic pathway.

10.1128/mBio.00007-21.3 FIG S3 ^1^H nuclear magnetic resonance spectroscopy for metabolites purified from liquid culture filtrates of the Δ*bcscd1* strain. Download FIG S3, TIF file, 0.7 MB. Copyright © 2021 Chen et al. 2021 Chen et al. https://creativecommons.org/licenses/by/4.0/ This content is distributed under the terms of the Creative Commons Attribution 4.0 International license.

10.1128/mBio.00007-21.4 FIG S4 Mass spectrum list of metabolites purified from the Δ*bcscd1* strain. Download FIG S4, TIF file, 1.0 MB. Copyright © 2021 Chen et al. 2021 Chen et al. https://creativecommons.org/licenses/by/4.0/ This content is distributed under the terms of the Creative Commons Attribution 4.0 International license.

**FIG 3:**
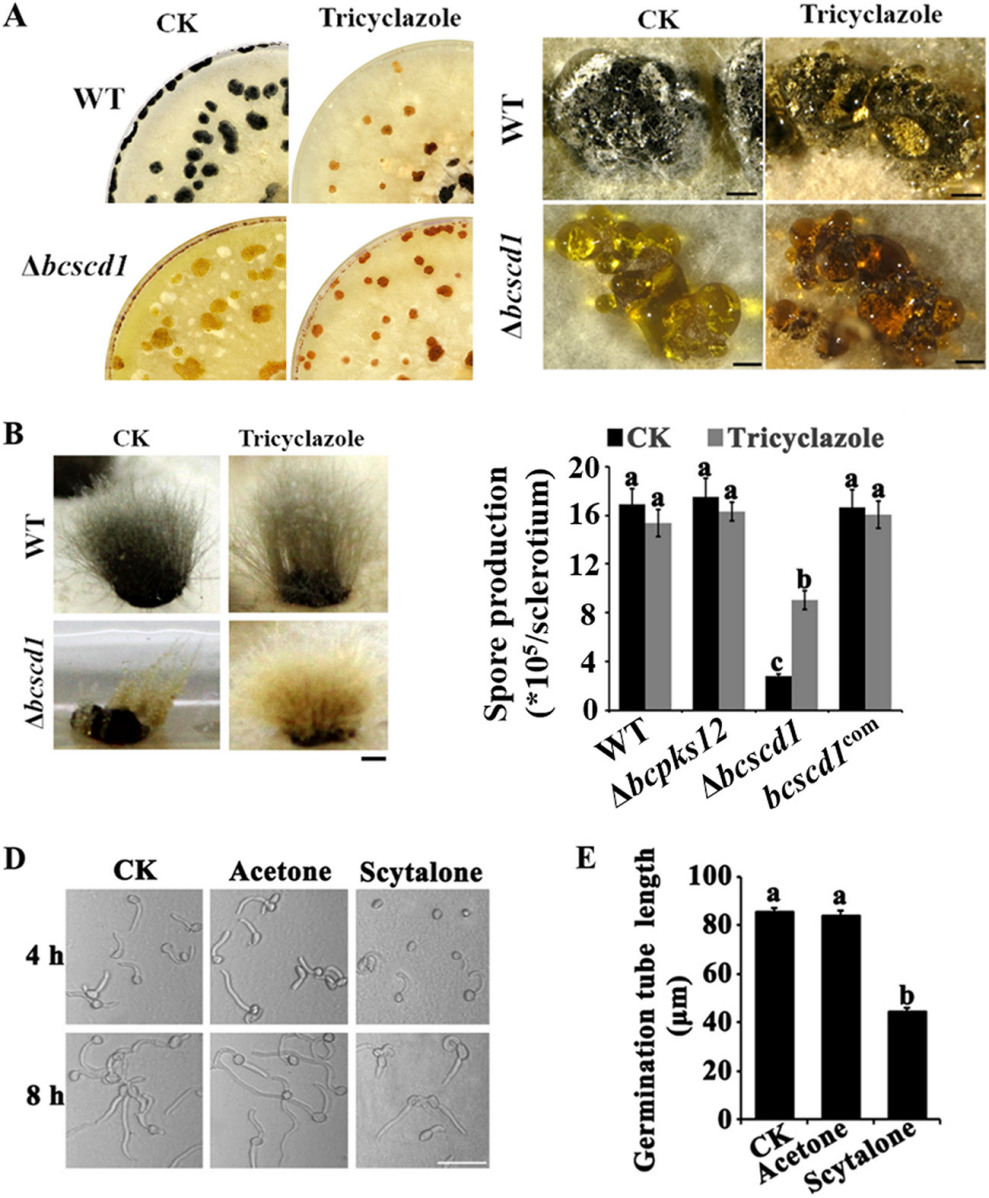
The melanin intermediate scytalone deposited by the Δ*bcscd1* mutant is inhibitory for sclerotial and spore germination. (A) The sclerotial color of WT and Δ*bcscd1* strains was altered when treated with the chemical inhibitor tricyclazole (50 μg/ml), which is supposed to inactivate the activity of the reductase BcBRN1/2 (bars, 1 mm). (B) Treatment with tricyclazole derepressed sclerotial germination of the Δ*bcscd1* mutant compared with the control group (CK) but caused no obvious effect on sclerotial germination of the WT (bar, 1 mm). (C) Spores produced by germinated sclerotia of the Δ*bcscd1* mutant were significantly reduced compared with the WT, Δ*bcpks12*, and complementation strains, and tricyclazole treatment increased the spore quantity produced by Δ*bcscd1* sclerotia but caused no effect on the other strains. (D and E) Feeding assay by the addition of scytalone (400 μg/ml) reduces spore germination and germ tube growth rates. CK, control check.

To investigate whether scytalone represses sclerotium germination and sporulation in the Δ*bcscd1* mutant, the enzymatic activity of BRN1 and -2 (BRN1/2), which reduce T4HN to scytalone, was blocked using the inhibitor tricyclazole (10 μg/ml) during sclerotial germination. The results showed that in the wild-type and Δ*bcpks12* strains, treatment with tricyclazole caused a color change in the mycelium and conidia generated from sclerotium germination but no significant change in the number of conidia produced by each sclerotium (Fig. 3A to C). Interestingly, the germination defect of Δ*bcscd1* sclerotia was partially restored by tricyclazole treatment (Fig. 3A to C). Since inactivating BcBRN1/2 by tricyclazole could lead to a decrease of scytalone biosynthesis in the Δ*bcscd1* mutant, it can be concluded that the sclerotium germination and sporulation defects of the Δ*bcscd1* mutant are caused by the accumulation of the melanin intermediate scytalone.

### Scytalone accumulation is self-inhibitory to *B. cinerea*.

**FIG 4:**
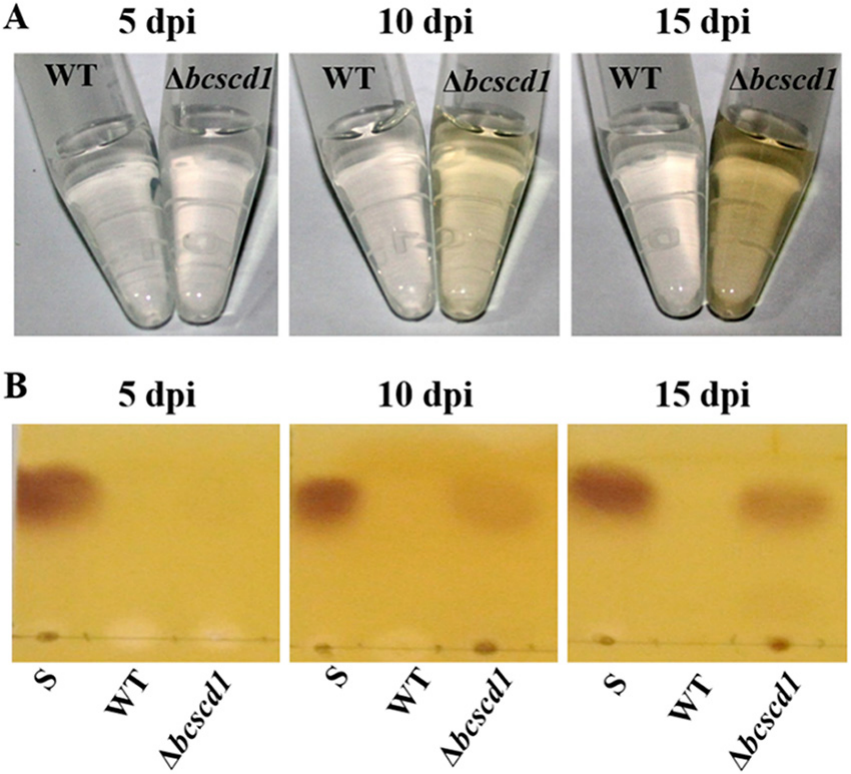
The melanin intermediate scytalone is accumulated in old but not young cultures of the Δ*bcscd1* mutant. (A) Pigmentation patterns of culture filtrates of the wild type (WT) and the Δ*bcscd1* mutant were compared at a series of growth stages. (B) TLC analysis of the crude extracts from culture filtrates of the wild type and the Δ*bcscd1* mutant at a series of growth stages. “S” represents the loading of standard scytalone.

To test whether scytalone causes an adverse effect on the growth and development of *B. cinerea*, we carried out spore germination assays in the presence of scytalone and found that 400 μg/ml scytalone was sufficient to suppress spore germination and germ tube elongation (Fig. 3D and E). Thus, scytalone is inhibitory to *B. cinerea*. However, a discrepancy with this inhibition is that in the Δ*bcscd1* mutant, there is no evidence for a defect in vegetative mycelium growth. To address this issue, TLC assays were conducted to analyze the scytalone accumulation levels of the wild-type and Δ*bcscd1* mutant strains at different growth stages (Fig. 4). The results showed that scytalone could not be detected in the Δ*bcscd1* mutant during early growth stages (before 5 days postinoculation [dpi]), when the mycelium color appeared white as for the wild type (Fig. S2A). As the cultures increased pigmentation during maturation (10 dpi), the extract from the Δ*bcscd1* culture showed scytalone production via TLC analysis, and scytalone accumulated further when cultures aged (15 dpi). In contrast, scytalone was not detected in the extracts of the wild type throughout the tested growth stages. Therefore, the Δ*bcscd1* strain showed a normal vegetative mycelium expansion rate during early growth stages and pathogenicity in host plants. In older cultures such as sporulating colonies and matured sclerotia, excessive scytalone is likely to cause cytotoxic effects, leading to defects in reproduction development.

### The scytalone-synthesizing enzymes BcBRN1/2 are localized in subcellular vesicles and trafficked to the cell wall.

**FIG 5:**
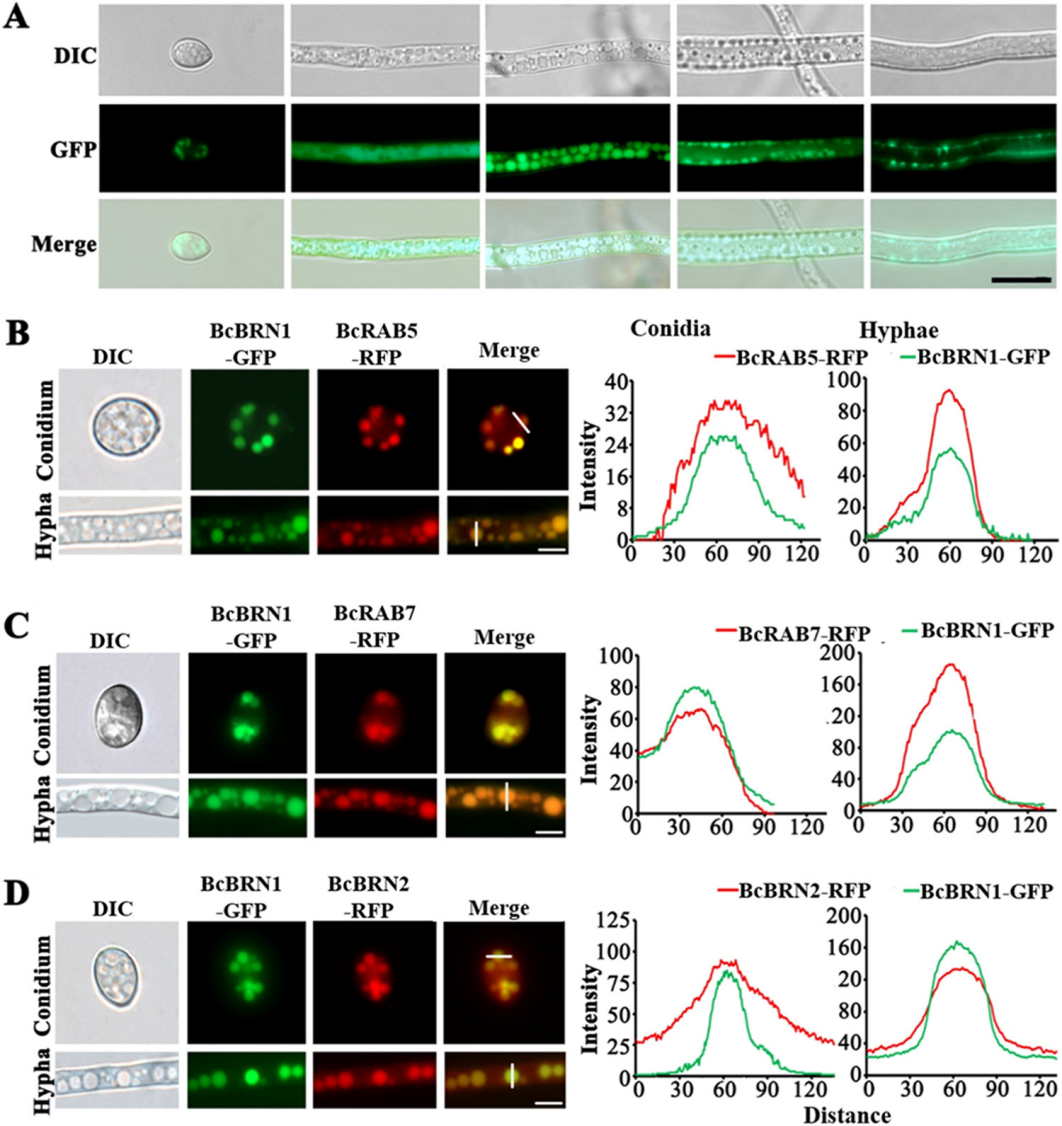
Localization of the reductases BcBRN1/2. (A) The fluorescence signal of GFP-labeled BcBRN1 in the WT+BcBRN1-GFP strain appeared at various subcellular sites in conidia and hyphae at different growth stages. DIC, differential interference contrast. (B to D) Simultaneous detection of GFP and RFP fluorescence in conidia and hyphae of the WT+BcBRN1-GFP+BcRAB5-RFP (B), WT+BcBRN1-GFP+BcRAB7-RFP (C), and WT+BcBRN1-GFP+BcBRN2-RFP (D) strains. Line scan graphs were generated at the indicated positions to show the relative localization of GFP (green) and RFP (red) signals. Conidia and hyphae were photographed 0 and 2 days after incubation, respectively. Bars, 10 μm (A) and 5 μm (B to D).

As scytalone accumulation is toxic to *B. cinerea*, our subsequent study focused on where scytalone is synthesized and how scytalone is delivered extracellularly. In the *B. cinerea* genome, there are two homologue genes, *Bcbrn1* and *Bcbrn2*, both encoding 1,3,6,8-tetrahydroxynaphthalene (T4HN) reductases that are responsible for synthesizing scytalone (29). The subcellular localization of BcBRN1 was first examined using the WT+BcBRN1-GFP strain, in which BcBRN1 being tagged with GFP was expressed in the wild-type background. Microscopy analysis revealed that in germinating conidia, the BcBRN1-GFP signal was detected in a limited number of vesicle structures inside the cytoplasm. In the elongating hyphae, however, fluorescence signals were observed in the following circumstances: (i) throughout the cytoplasm in the hyphae, (ii) in randomly distributed punctate structures (mean diameter of 2.07 ± 0.07 μm [*n* = 120]), (iii) in smaller punctate structures (mean diameter of 1.54 ± 0.07 μm [*n* = 120]) and arranged along the inner sides of the hyphal lumen, and (iv) fused with the hyphal periphery (Fig. 5A). These results suggest that the subcellular localization of BcBRN1 is dynamic, implicating trafficking from the cytoplasm to the cell surface via vesicles.

To determine whether BcBRN1-labeled vesicles represent endosomes from the endomembrane system (32), two endosome-specific proteins of *B. cinerea* (BcRAB5 and BcRAB7) were fused with red fluorescent protein (RFP) to test whether they colocalized with BcBRN1-GFP. Consequently, WT+*Bcbrn1-GFP*+*Bcrab5-RFP* and WT+*Bcbrn1-GFP*+*Bcrab7-RFP* strains were obtained and further analyzed. In both conidia and hyphae, BcBRN1-GFP appeared at almost the same sites as BcRAB5-RFP and BcRAB7-RFP, and line scan analyses of the fluorescence signal intensities indicated that the relative localizations of GFP (green) and RFP (red) signals well overlapped each other (Fig. 5B and C). Moreover, as BcBRN1 and BcBRN2 are homologous proteins involved in DHN melanin synthesis in *B. cinerea*, the WT+*Bcbrn1-GFP*+*Bcbrn2-RFP* strain was observed by fluorescence microscopy. Figure 5D shows that the BcBRN1-GFP and BcBRN2-RFP signals colocalized in the same intracellular vesicle structures. To rule out GFP signal bleed-through in the RFP channel for the dually tagged strains, we constructed strains harboring either GFP (WT-*GFP*) or RFP (WT-*RFP*) and detected the fluorescence signal in dual channels. In elongating hyphae, no RFP signals were visualized for the WT-*GFP* strain, and no GFP signals were observed for the WT-*RFP* strain (Fig. S5). Therefore, the colocalizations between BcBRN1/2 and the endosome markers were authentic.

10.1128/mBio.00007-21.5 FIG S5 Fluorescence microscopic analysis for ruling out autofluorescence of the fungus and for detecting possible bleed-through between different fluorescent marker proteins. (A) WT+*GFP* and WT+*RFP* strains observed under DIC, GFP, and RFP channels. (B) Microscopic observation of the transformants with BcRAB5-RFP, BcRAB7-RFP, or BcPEX3-RFP alone under DIC, GFP, and RFP channels. Bars, 50 μm. Download FIG S5, TIF file, 0.6 MB. Copyright © 2021 Chen et al. 2021 Chen et al. https://creativecommons.org/licenses/by/4.0/ This content is distributed under the terms of the Creative Commons Attribution 4.0 International license.

**FIG 6:**
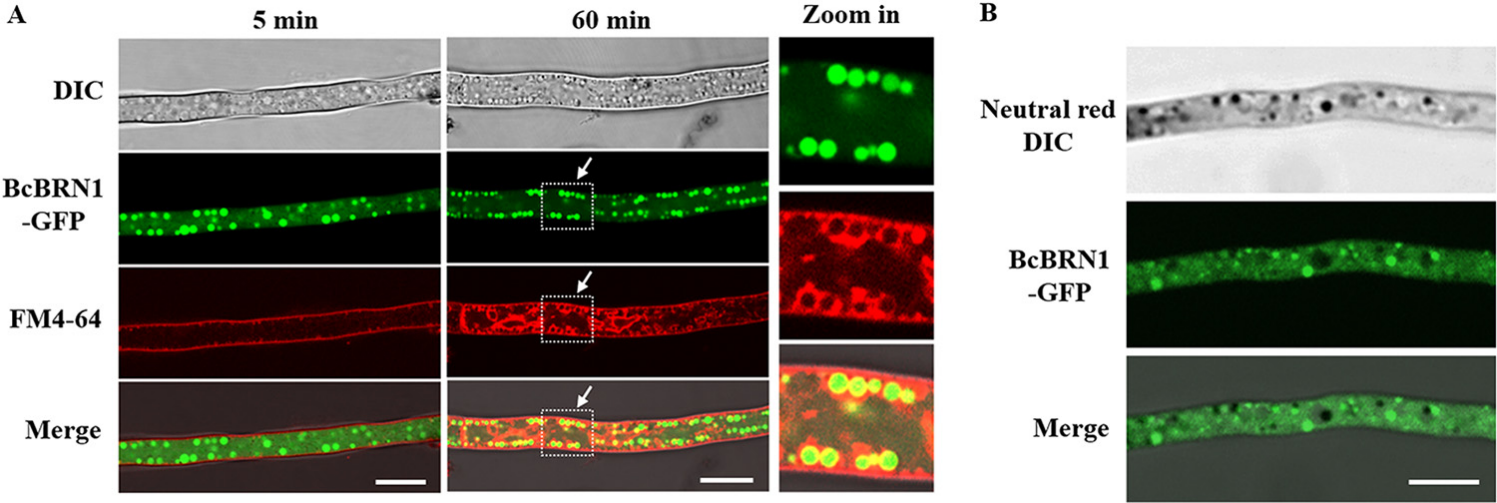
Relative localization analysis with BcBRN1-GFP and FM4-64 or neutral red staining signals. (A) The endocytic pathway was traced using FM4-64 in the WT+BcBRN1-GFP strain. Staining periods in minutes are given at the top. The dye entered the plasma membranes (5 min), and subsequently, plasma membrane-conglutinated endocytic vesicles were observed (60 min). Close views of the dotted rectangle areas indicated by arrows are shown on the right side. In the hyphae, BcBRN1-GFP is localized within the spherical vesicles, which can be partially colocalized with the endocytic vesicles. (B) Visualization of BcBRN1-GFP fluorescence and neutral red staining signals in the WT+BcBRN1-GFP strain. Vacuoles stained with neutral red and observed by DIC microscopy (black) are distinguishable from the vesicles localized with BcBRN1-GFP. Bars, 10 μm.

Moreover, staining assays with specific dyes indicated that the vesicles labeled with BcBRN1-GFP were spherical and colocalized with the FM4-64 fluorescence signal, which is indicative of endosome vesicles (Fig. 6A). Additionally, the BcBRN1-GFP-labeled subcellular spaces were clearly distinguishable from the vacuoles, which were stained with neutral red (Fig. 6B).

### Scytalone accumulation leads to an increased number of BcBRN1/2-labeled vesicles in the cytoplasm.

**FIG 7:**
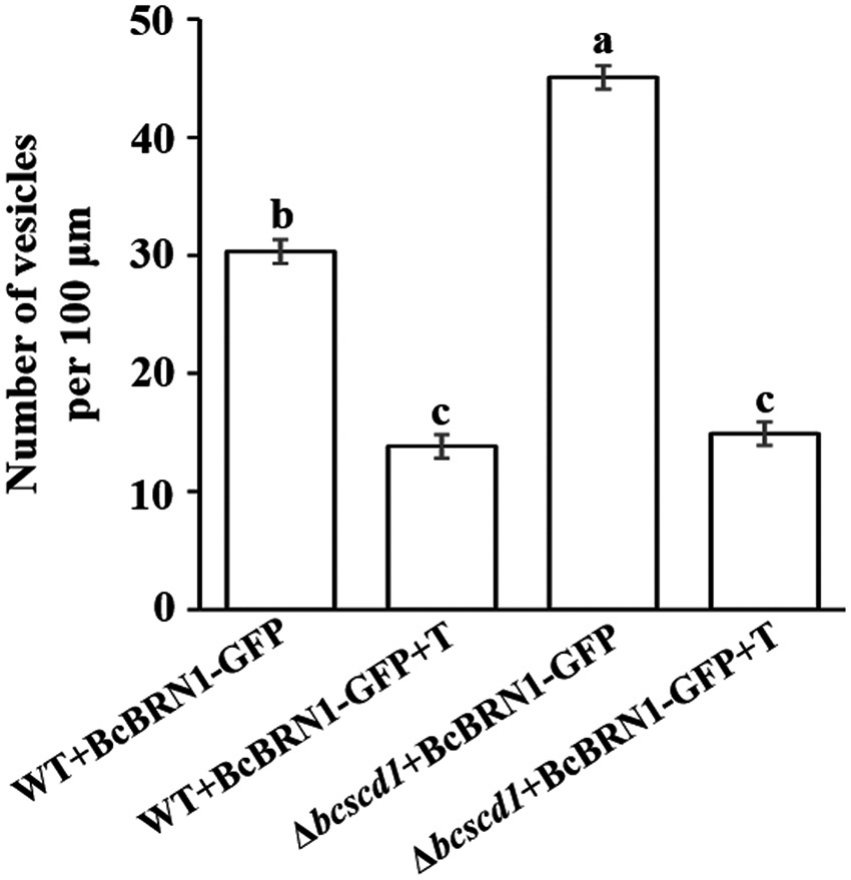
Effect of scytalone accumulation on the density of BcBRN1-GFP-labeled vesicles. Shown are the changes of BcBRN1-GFP-labeled vesicles in mycelia of the WT+BcBRN1-GFP and Δ*bcscd1*+BcBRN1-GFP strains in the presence or absence of tricyclazole (T) (10 μg/ml). Different letters represent significant differences between columns (*n* = 150; *P* < 0.05).

As the intermediate scytalone is secreted outside the cells, the number and distribution of vesicles containing the scytalone synthesis enzyme BcBRN1 were further analyzed. Considering that the deletion of *Bcscd1* resulted in the accumulation of scytalone, constructs expressing BcBRN1-GFP driven by the native promoter were transfected into both the wild-type and Δ*bcscd1* strains to trace the change in the number of BcBRN1-GFP-labeled vesicles. In the WT+*Bcbrn1-GFP* strain, the vesicles showing a BcBRN1-GFP signal were distributed in the mycelia, with an average density of 30 fluorescent vesicles per 100 μm hyphae (Fig. 7). In contrast, the BcBRN1-GFP-marked vesicles in mycelia of the Δ*bcscd1* mutant were even more densely distributed, with an average of 46 fluorescent vesicles per 100 μm hyphae. Moreover, upon treatment with 10 μg/ml tricyclazole, the fluorescent vesicles were sharply reduced in both the WT+*Bcbrn1-GFP* and Δ*bcscd1*+*Bcbrn1-GFP* strains to similar levels (about 12 to 15 per 100 μm hyphae) (Fig. 7). These data suggested that more BcBRN1/2-labeled vesicles in the Δ*bcscd1* mutant are associated with its extracellular secretion of the intermediate product scytalone.

### The localization of other melanin synthetic enzymes defines distinctive subcellular compartmentalization and trafficking in *B. cinerea*.

**FIG 8:**
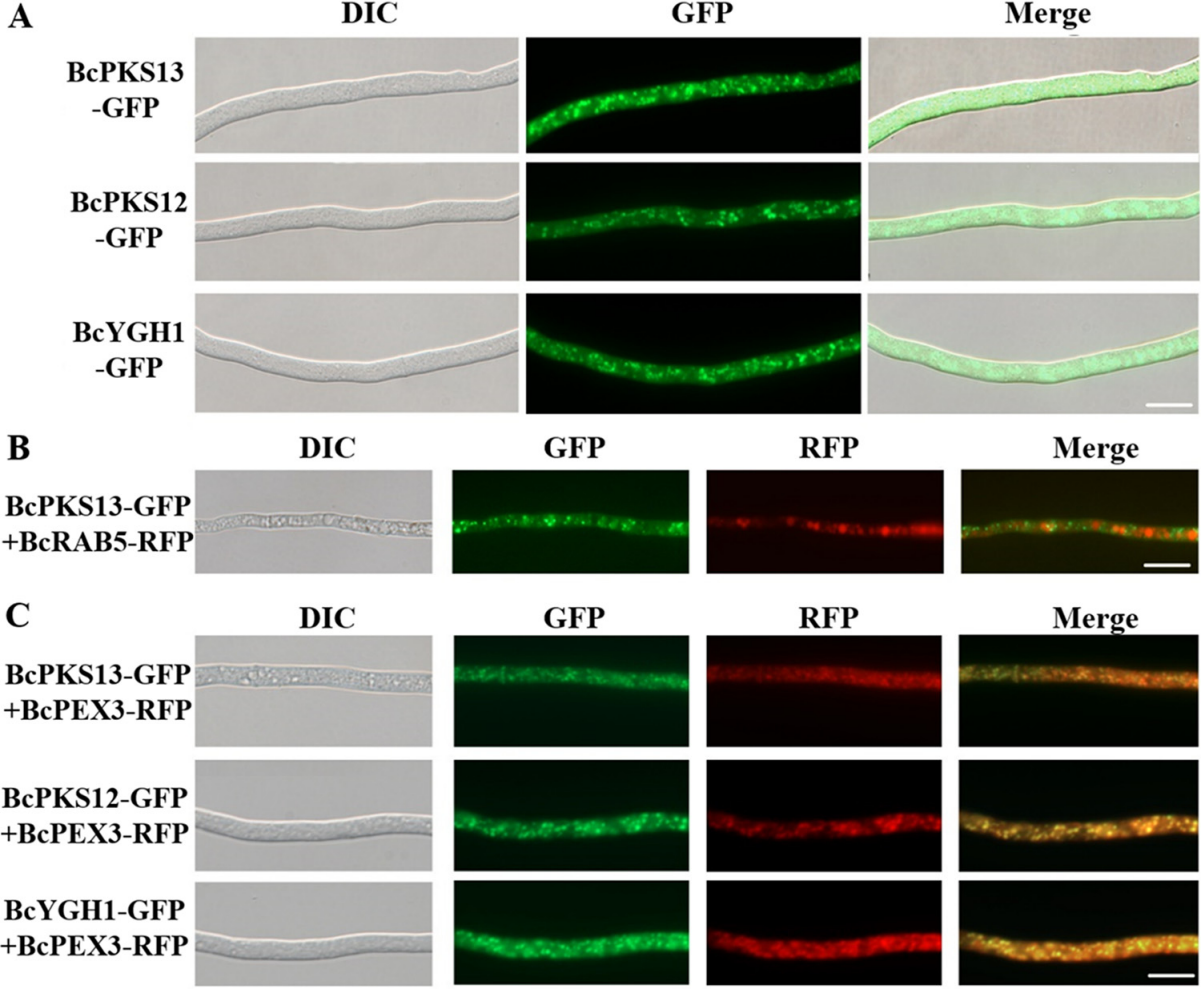
Subcellular localization of enzymes at the early steps of melanin synthesis. (A) Subcellular localization of BcPKS12, BcPKS13, and BcYGH1 in the hyphae of the WT+BcPKS12-GFP, WT+BcPKS13-GFP, and WT+BcYGH1-GFP strains, respectively. (B) Relative localization of BcPKS13 and BcRAB5 in the WT+BcPKS13-GFP+BcRAB5-RFP strain. (C) The peroxisome membrane protein BcPEX3 colocalizes with BcPKS12, BcPKS13, and BcYGH1 in the hyphae of the WT+BcPKS12-GFP+BcPEX3-RFP, WT+BcPKS13-GFP+BcPEX3-RFP, and WT+BcYGH1-GFP+BcPEX3-RFP strains, respectively. Bars, 10 μm.

The polyketide synthases BcPKS12/13 and the hydrolase BcYGH1 have been defined as upstream steps of the melanin biosynthetic pathway (29). To investigate the subcellular localization of BcPKS12/13 and BcYGH1, we generated transgenic strains in which BcPKS13-GFP, BcPKS12-GFP, and BcYGH1-GFP were individually driven by their own promoters and expressed in the WT background. In the mycelia of these strains, the fluorescence signals were localized in small granular vesicles, with an average diameter of 0.87 ± 0.04 μm (*n* = 120) (Fig. 8A). When BcPKS13-GFP was coexpressed with BcRAB5-RFP, vesicles labeled with BcPKS13-GFP were overall smaller than those labeled with BcRAB5-RFP, and these two fluorescence signals could be clearly distinguished (Fig. 8B). Since BcBRN1/2 have been demonstrated to colocalize with the endosome markers (BcRAB5 and BcRAB7), these data thus suggest that the vesicles carrying BcPKS13 are different from the BcBRN1/2-labeled endosomes.

Peroxisomes of fungi are involved in various secondary metabolite synthesis pathways (33). For example, the basic building block for the formation of fungal polyketides, acetyl-CoA, can be formed by β-oxidation of fatty acids in peroxisomes (34). In order to verify whether peroxisomes are involved in melanin synthesis, the peroxisome membrane protein BcPEX3 was tagged with RFP and expressed in the background of the WT-BcPKS12-GFP, WT-BcPKS13-GFP, and WT-BcYGH1-GFP strains. Figure 8C shows that the peroxisomes labeled with BcPEX3-RFP in mycelia overlapped the subcellular localization of BcPKS13-GFP, BcYGH1-GFP, and BcPKS12-GFP fluorescence signals, indicating that these enzymes for early steps of melanin synthesis are enclosed in peroxisomes.

**FIG 9:**
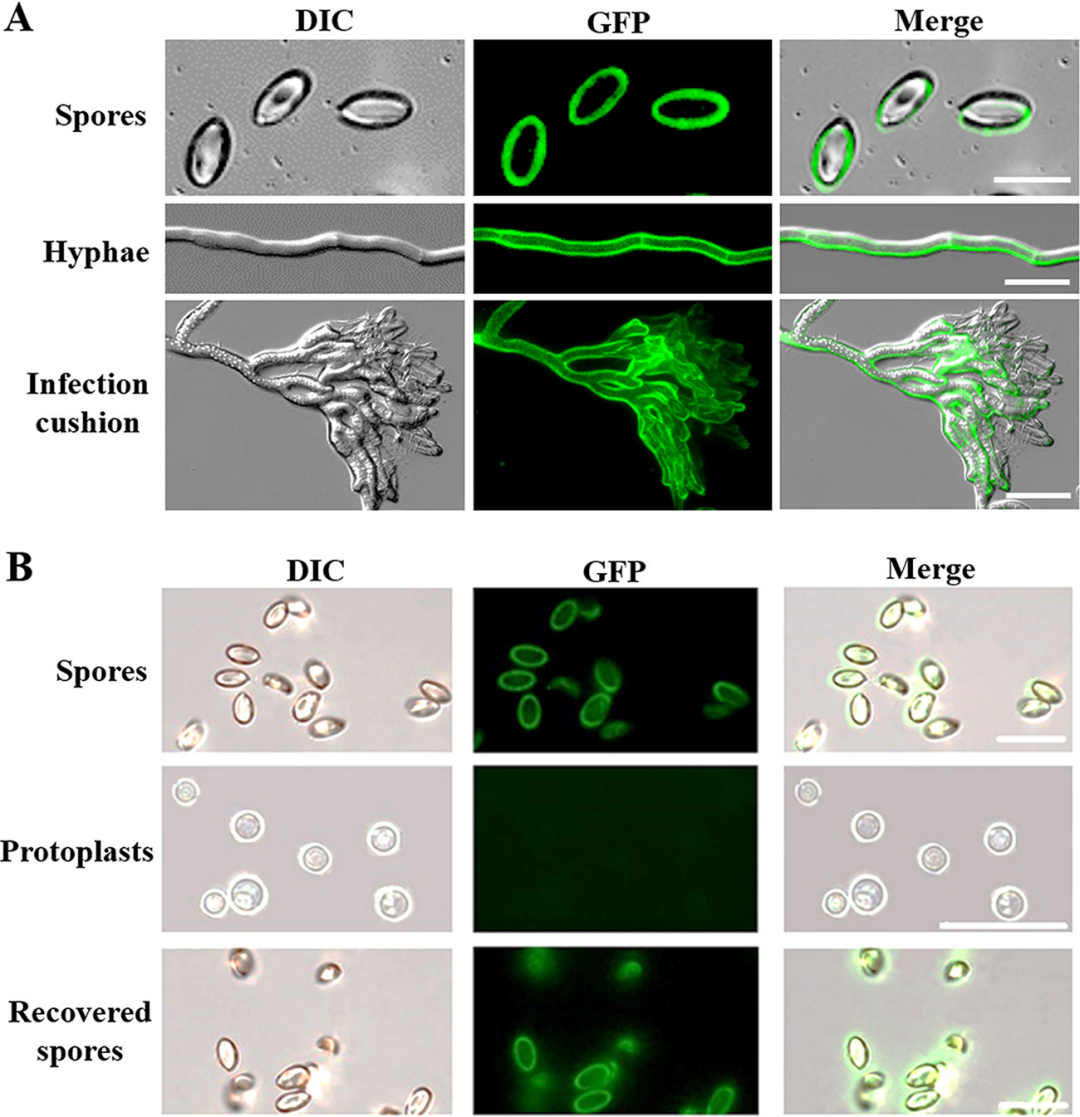
(A) Light and fluorescence microscopic views of spores, hyphae, and infection cushions of the *bcscd1*^com^ (Δ*bcscd1*+BcSCD1-GFP) strain. (B) Spores of the *bcscd1*^com^ strain were subjected to cell wall digestion and regeneration assays. The samples for analysis included spores, protoplasts, and recovered spores obtained from the regenerated culture of protoplasts. Bars, 10 μm (A) and 20 μm (B).

**FIG 10:**
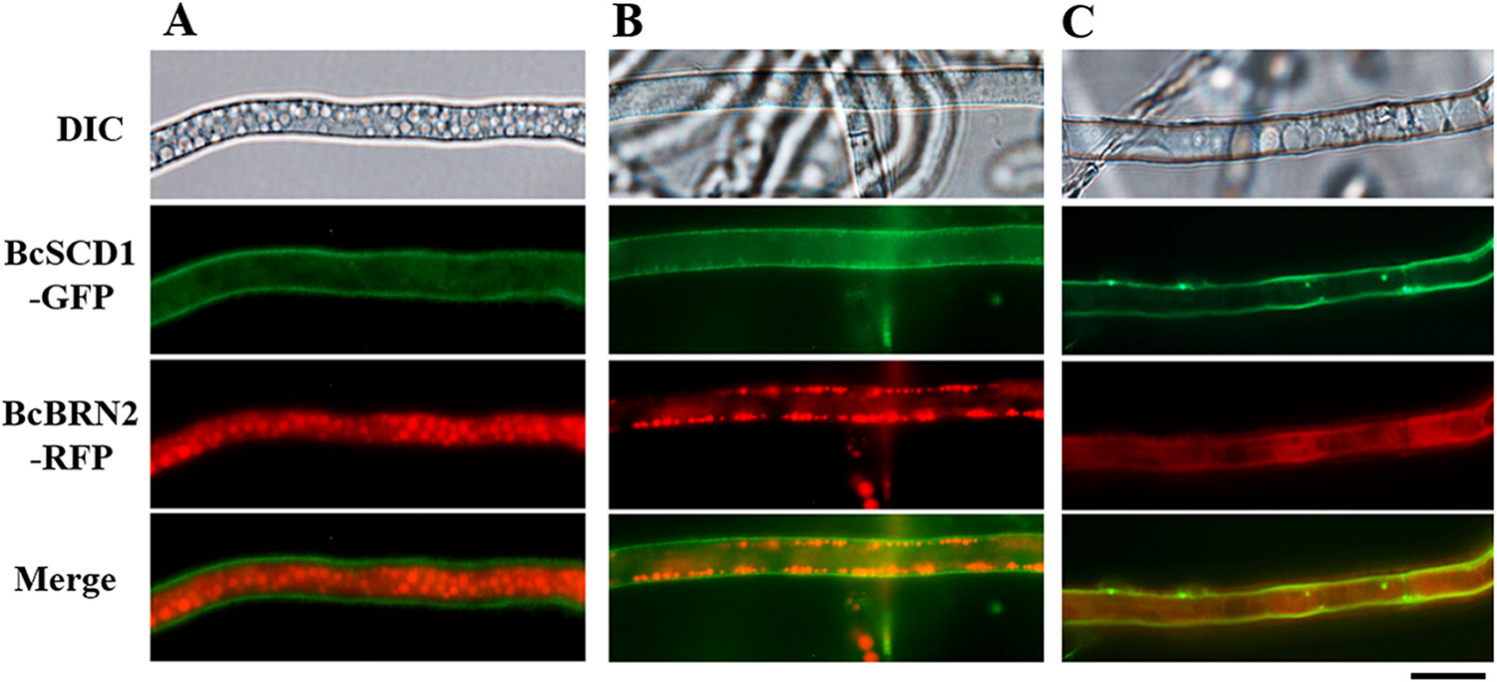
Subcellular localization of BcSCD1-GFP and BRN2-RFP. Simultaneous detection of GFP-labeled BcSCD1 and RFP-labeled BcBRN2 was conducted in growing hyphae of the Δ*bcscd1*+BcSCD1-GFP+BRN2-RFP strain. (A and B) BRN2-RFP signals are present in the cytoplasm and subcellular vesicles, being distinguished from the location of the BcSCD1-GFP signal on the cell wall. (C) BRN2-RFP and BcSCD1-GFP are colocalized on the cell wall. Bar, 10 μm.

Using the Δ*bcscd1*+*Bcscd1-GFP* strain, the subcellular localization of BcSCD1 was also analyzed. Figure 9A shows positive BcSCD1-GFP signals around the surfaces of conidia, hyphae, and infection cushions. To clarify whether BcSCD1-GFP is located on the cell wall or cell membrane, cell wall digestion and regeneration treatments of conidia were conducted. As shown in Fig. 9B, the BcSCD1-GFP fluorescence of conidia disappeared after their cell wall was digested, and the fluorescence signals were recovered after the protoplasts had regenerated the cell walls. From these data, it can be concluded that BcSCD1-GFP is localized in the cell wall and not the cell membrane. Additionally, the localization relationship between GFP-labeled BcSCD1 and RFP-labeled BcBRN2 was analyzed using the strain carrying these two fluorescently labeled proteins. The BcSCD1-GFP and BcBRN2-RFP signals were not overlapping in the cytoplasm, but they colocalized on the cell wall (Fig. 10). These findings suggest that after the intermediate scytalone is transported to the cell wall, the subsequent downstream reactions catalyzed alternately by BcSCD1 and BcBRNs in the pathway should occur on the cell surface.

## DISCUSSION

As a DHN melanin-producing fungus, *B. cinerea* has a nonlinear DHN melanogenesis pathway, with two polyketide synthases (PKSs), BcPKS12 and BcPKS13, which are involved in melanogenesis in sclerotia and conidia, respectively. However, the pathway downstream of the PKSs is shared in both reproduction structures (29). Although the DHN biosynthetic pathway has been sketched in *B. cinerea*, where the enzymes involved in this pathway function and how DHN melanin is transported to the cell wall are open questions.

The established DHN melanin synthesis pathway in *B. cinerea* indicates that BcSCD1 is responsible for the conversion of scytalone to T3HN (29). This study revealed that the Δ*bcscd1* mutant decreased conidium production and sporogenesis germination of sclerotia. Mutation of *Bcscd1* probably leads to the accumulation of scytalone in the fungal culture. As expected, a compound abundantly produced by the Δ*bcscd1* mutant was identified as scytalone by NMR and mass spectrometry analyses. However, scytalone was extracted only from the culture filtrate but not from the mycelium biomass, suggesting that scytalone produced by the Δ*bcscd1* mutant is mainly secreted to the extracellular space. The coculture assay with the Δ*bcscd1* and Δ*bcpks12* mutants showed that the Δ*bcpks12* sclerotia were gradually melanized from the borderline between the two colonies, further validating that scytalone is secreted extracellularly. Functional analysis of the scytalone dehydratase orthologue in the anthracnose pathogen Colletotrichum gloeosporioides also supports the notion that the intermediate scytalone is secreted extracellularly for further melanin synthesis (35).

Besides being an intermediate for DHN melanin synthesis, scytalone has additional functions. It has been reported that scytalone could cause toxic effects on grape leaves, lettuce roots, and hypocotyls (36, 37). On the other hand, scytalone promoted the growth of Arabidopsis thaliana root and grape calluses (38). This study revealed that exogenously applied scytalone suppressed the germination of wild-type spores, while treatment with tricyclazole, which is a specific inhibitor of BcBRN1/2 upstream of BcSCD1 in the DHN melanin synthesis pathway (39), largely restored the germination of Δ*bcscd1* sclerotia. These results suggest that scytalone is cytotoxic, and its accumulation in the Δ*bcscd1* culture is responsible for suppressing sclerotial germination and conidiation.

The biosynthesis of fungal secondary metabolites such as penicillin, aflatoxin, trichothecenes, and deoxynivalenol (DON) toxins can be conducted in vesicular compartments to avoid their self-toxicity (40). Given that the melanin intermediate scytalone is self-toxic, enzymes for melanin synthesis could be compartmentalized at certain subcellular sites. Indeed, enzymes involved in different steps of the biosynthesis of melanin are targeted to multiple subcellular locations in *B. cinerea*. The polyketide synthases BcPKS12/13 and the hydrolase BcYGH1 that act on the conversion of the precursor acetyl-CoA into T4HN for the early steps of melanin synthesis were localized in the BcPEX3-RFP-labeled peroxisomes. This is practicable as the peroxisomal β-oxidation of fatty acids results in the formation of acetyl-CoA, which is the starting precursor for the biosynthesis of fungal polyketides (34), in accordance with the findings that functional peroxisomes and peroxisomal acetyl-CoA are essential for appressorial melanin synthesis and host invasion by the rice blast fungus Magnaporthe grisea (41). Moreover, the involvement of peroxisomes in secondary metabolism is common in fungi, including aflatoxin production in Aspergillus parasiticus (40), penicillin production in Penicillium chrysogenum (42), and aurofusarin (43) and DON toxin (44) production in Fusarium graminearum.

The subsequent product of BcPKS12/13 and BcYGH1, T4HN, is catalyzed by the T4HN reductases BcBRN1/2, leading to the intermediate scytalone (29). BcBRN1 and BcBRN2 were shown to be closely colocalized in the cytoplasm and appeared in membrane-enclosed vesicular structures stained by the fluorescent dye FM4-64. Furthermore, these vesicles are indicative of endosomes due to the colocalization of BcBRN1-GFP with the endosome markers BcRAB5-RFP and BcRAB7-RFP. Similarly, it is reported that the homologue of T4HN reductase, the Arp2 of A. fumigatus, also locates in endosomes (32).

Interestingly, melanin and melanogenesis in mammals are confined to a membrane-bound organelle called a melanosome, which is a lysosome-related organelle and is hypothesized to help sequester toxic intermediates and prevent oxidative stress (45, 46). Thus, fungal melanin may be synthesized in internal vesicles akin to mammalian melanosomes and transported to the cell wall. A recent study reveals that the DHN pathway enzymes of Neurospora crassa are also localized to undetermined nonuniform intracellular vesicles (47). Our studies uncovered that the biosynthesis of melanin involves enzymes targeted to multiple subcellular locations, including peroxisomes and endosomes. This phenomenon is reminiscent of a report in Aspergillus nidulans that peroxisomes are tethered with early endosomes via the coiled-coil-domain-containing protein PxdA (48). Considering the cytotoxic effect of scytalone produced by BcBRN1 and BcBRN2, it is reasonable to assume that scytalone and its producing enzymes may be colocalized within the same membranous vesicles, thus providing protection for the cell.

Additionally, we observed the dynamic subcellular localizations of BcBRN1-GFP and BcBRN2-RFP. This changing localization pattern of BcBRN1 and BcBRN2 is attributed to their actions on two steps in melanin synthesis, required for the early catalytic step that converts T4HN to scytalone and the later step that converts T3HN to vermelone. Downstream of BcBRN1 and BcBRN2 is the secreted dehydratase BcSCD1 that is responsible for catalyzing the steps of the conversion of scytalone to T3HN and vermelone to 1,8-DHN. According to the subcellular location of enzymes for melanin synthesis and the cytotoxic effect of scytalone, melanin synthesis can apparently be divided into intracellular and extracellular synthetic processes. The intracellular stage includes all the steps until the intermediate scytalone is transported to the cell surface, whereas the extracellular process encloses all the steps after scytalone production.

**FIG 11:**
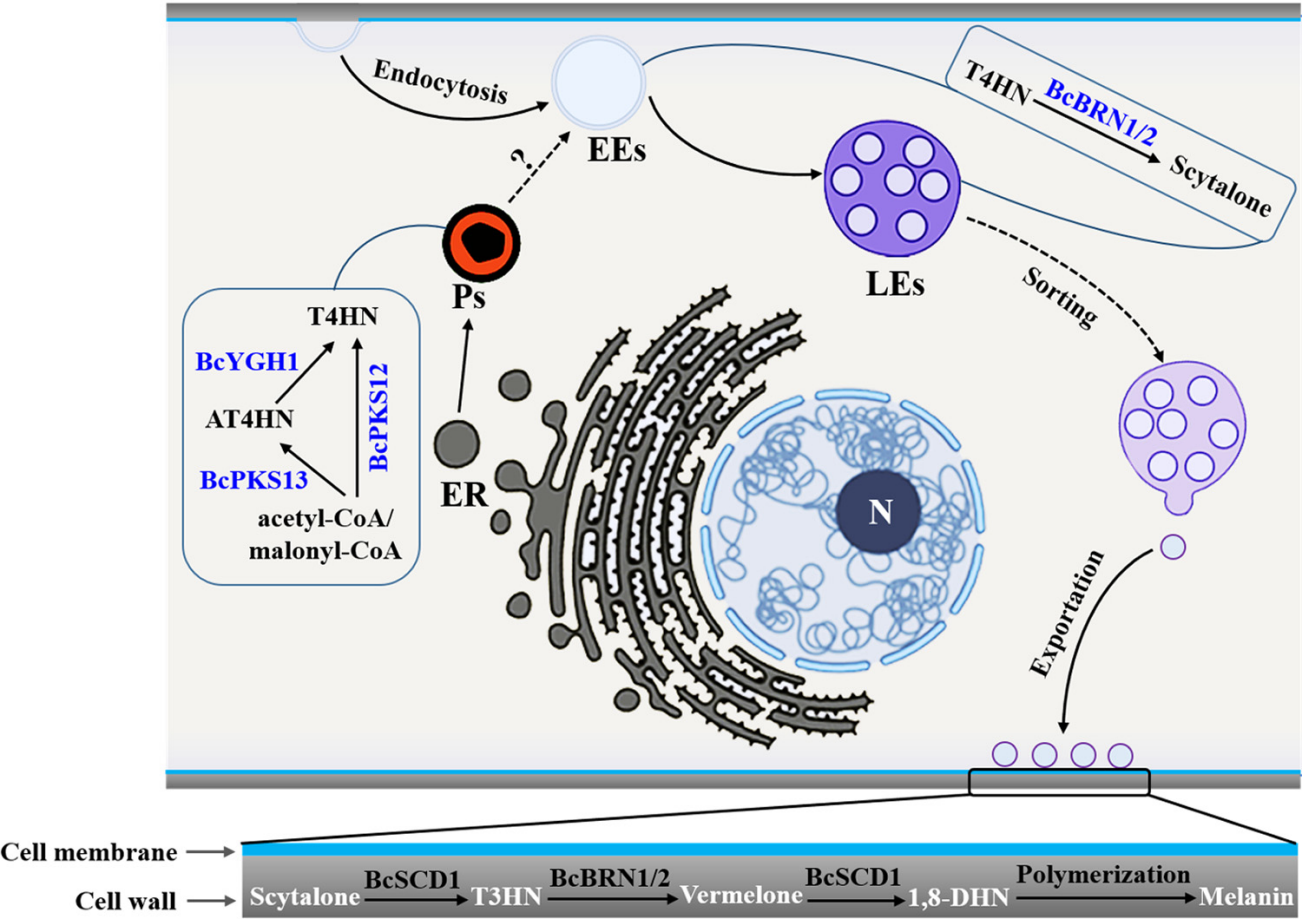
Cytological regulation model for melanin synthesis in *B. cinerea*. N, nucleus; ER, endoplasmic reticulum; Ps, peroxisome; EEs, early endosomes; LEs, late endosomes; AT4HN, 2-acetyl-1,3,6,8-tetrahydroxynaphthalene.

In summary, the present study addresses the significance of melanin synthesis in the life cycle of *B. cinerea* and presents general subcellular locations for melanin synthesis in this fungus (Fig. 11), primarily demonstrating that the early polyketide synthase and hydrolase steps are located to the peroxisomes, which are subsequently connected with the actions of T4HN reductases in endosomes. The product of T4HN reductases, scytalone, is by unknown mechanisms delivered to the extracellular space (cell wall) for subsequent synthesis reactions, most likely to avoid the toxic effects of scytalone on fungal cells. Consequently, the steps after scytalone are located on the cell wall, leading to the accumulation of the final melanin products there.

## MATERIALS AND METHODS

### Fungal strains and growth conditions.

The *B. cinerea* strain B05.10 was used as the wild type and as the recipient for mutant strain construction. The wild-type and mutant strains were cultured on complete medium (CM) (that is, 30 g sucrose, 1 g KH_2_PO_4_, 0.5 g MgSO_4_·7H_2_O, 0.5 g KCl, 2 g NaNO_3_, 2.5 g N-Z amine, 1 g yeast extract,10 ml vitamin stock solution, and 0.2 ml trace element solution in 1 liter of water, with 20 g agar for solid medium) at 23°C under light illumination for sporulation and in the dark for sclerotium formation. Yeast-sugar-salt (YSS) medium [2 g of yeast extract, 10 g of glucose, 2 g of KH_2_PO_4_, 1.5 g of K_2_HPO_4_, 1 g of (NH_4_)_2_SO_4_, and 0.5 g of MgSO_4_·7H_2_O per liter] was used for culturing to obtain melanin intermediate metabolites (49).

### Generation of transgenic fungal mutant strains.

The gene deletion cassette was constructed as follows. First, the 5′- and 3′-flanking regions (∼1 kb) of the target gene were amplified from genomic DNA of the wild type using sequence-specific primer pairs. The amplified fragments were fused with the hygromycin (Hyg) resistance gene (*hyg*) of pCAMBI1300 by fusion PCR (Fig. S6A in the supplemental material demonstrates an example of generating the *Bcscd1* deletion cassette). The transformation was mediated by polyethylene glycol on protoplasts, as previously reported for site-specific homologous recombination (50). The mutant obtained was identified by diagnostic PCR using specific primer pairs (Fig. S7). The primer pairs used are listed in Table S1.

10.1128/mBio.00007-21.6 FIG S6 Schematic representation of constructing *bcscd1* deletion and fluorescence-labeled mutants of *B. cinerea*. (A) Homologous recombination strategy for constructing the *bcscd1* deletion mutant (Δ*bcscd1*). (B) pNR2 vector used for GFP labeling of *bcpks12*, *bcpks13*, *bcygh1*, and *bcbrn1* gene products. (C) pNDF-OCT vector used for RFP labeling of *bcrab5*, *bcrab7*, and *bcbrn2* gene products. Download FIG S6, TIF file, 0.5 MB. Copyright © 2021 Chen et al. 2021 Chen et al. https://creativecommons.org/licenses/by/4.0/ This content is distributed under the terms of the Creative Commons Attribution 4.0 International license.

10.1128/mBio.00007-21.7 FIG S7 Electrophoretic bands of target fragments PCR amplified from chromosomal DNAs of the mutants constructed in the present work. (A) Δ*bcscd1* (lane 1), wild type (WT) (lane 2), and water (negative control) (lane 3). (B) Δ*bcscd1*+BcSCD1-GFP (*bcscd1*^com^) (lane 4), pNR2-BcSCD1-GFP (positive control) (lane 5), and water (negative control) (lane 6). (C) Δ*bcscd1*+BcBRN1-GFP (lane 7), pNR2-BcBRN1-GFP (positive control) (lane 8), and water (negative control) (lane 9). (D) Δ*bcscd1*+BcSCD1-GFP+BcBRN2-RFP (lane 10), Δ*bcscd1*+BcSCD1-GFP (lane 11), and water (negative control) (lane 12). (E) WT+GFP (lane 13), pNR2-GFP (positive control) (lane 14), and WT (negative control) (lane 15). (F) WT+BcBRN1-GFP (lane 16), pNR2-BcBRN1-GFP (positive control) (lane 17), and WT (negative control) (lane 18). (G) WT+BcBRN1-GFP+BcRAB5-RFP (lane 19), WT+BcBRN1-GFP (lane 20), and water (negative control) (lane 21). (H) WT+BcBRN1-GFP+BcRAB7-RFP (lane 22), WT+BcBRN1-GFP (lane 23), and water (negative control) (lane 24). (I) WT+BcBRN1-GFP+BcBRN2-RFP (lane 25), WT+BcBRN1-GFP (lane 26), and water (negative control) (lane 27). (J) WT+BcPKS13-GFP (lane 28), pNR2-BcPKS13-GFP (positive control) (lane 29), and WT (negative control) (lane 30). (K) WT+BcPKS13-GFP+BcRAB5-RFP (lane 31), WT+BcPKS13-GFP (lane 32), and WT (negative control) (lane 33). (L) WT+BcPKS13-GFP+BcPEX3-RFP (lane 34), WT+BcPKS13-GFP+BcPEX3-RFP (lane 35), and water (negative control) (lane 36). (M) WT+BcYGH1-GFP+BcPEX3-RFP (lane 37), WT+BcYGH1-GFP (lane 38), and water (negative control) (lane 39). (N) WT+BcYGH1-GFP (lane 40), pNR2-BcYGH1-GFP (positive control) (lane 41), and WT (negative control) (lane 42). (O) WT+BcPKS12-GFP (lane 43), pNR2-BcPKS12-GFP (positive control) (lane 44), and WT (negative control) (lane 45). (P) WT+BcPKS12-GFP+BcPEX3-RFP (lane 46), WT+BcPKS12-GFP (lane 47), and water (negative control) (lane 48). Target DNA fragments of these mutants were PCR amplified using specific primer pairs listed in Table S1 in the supplemental material. Download FIG S7, TIF file, 0.5 MB. Copyright © 2021 Chen et al. 2021 Chen et al. https://creativecommons.org/licenses/by/4.0/ This content is distributed under the terms of the Creative Commons Attribution 4.0 International license.

10.1128/mBio.00007-21.8 TABLE S1 Primer pairs used for target amplification by PCR. Download Table S1, DOCX file, 0.02 MB. Copyright © 2021 Chen et al. 2021 Chen et al. https://creativecommons.org/licenses/by/4.0/ This content is distributed under the terms of the Creative Commons Attribution 4.0 International license.

### Construction of fluorescence-labeled mutants.

To construct a *GFP-*labeled fusion cassette, the open reading frame (ORF) of the target gene (without the stop codon) together with its promoter region was amplified. The resulting product was assembled with the *GFP* gene in the SacI/XhoI-digested pNR2 plasmid fragment using a one-step cloning kit (Yeasen, China). Figure S6B in the supplemental material demonstrates an example of generating the *Bcscd1-GFP* fusion cassette. The recombinant plasmids were transferred into Escherichia coli DH5α competent cells, and ampicillin-resistant clones were screened by colony PCR and sequencing to obtain the correctly conjugated expression vector, which was subsequently transferred into the fungal protoplasts to obtain the strains with fluorescence-tagging signals. Similarly, some target genes were fused with the mCherry red fluorescent protein gene (*RFP*) in the plasmid pNDF-OCT (Fig. S6C) (51). Cotransformation with various combinations of the *RFP*-labeled and *GFP*-labeled cassettes into fungal protoplasts leads to the strains carrying two fluorescence-labeled proteins. All fluorescence-labeled strains used in this study are listed in Table 1. All these strains have been screened by PCR of target genes (Fig. S7) with the primers listed in Table S1.

**TABLE 1:** Fungal strains used in this study

Strain	Description
B05.10	Wild-type strain
Δ*bcpks12*	*Bcpks12* deletion mutant
Δ*bcscd1*	*Bcscd1* deletion mutant
Δ*bcscd1-*C-*GFP*	*Bcscd1* complementation mutant
WT+*Bcbrn1-GFP*	Expressing BcBRN1-GFP in B05.10
Δ*bcscd1*+*Bcbrn1-GFP*	Expressing BcBRN1-GFP in the Δ*bcscd1* mutant
Δ*bcscd1*-C-*GFP*+*Bcbrn2-RFP*	Expressing BcSCD1-GFP and BcBRN2-RFP in the Δ*bcscd1* mutant
WT+*Bcbrn1-GFP*+*Bcrab5-RFP*	Expressing BcBRN1-GFP and BcRAB5-RFP in B05.10
WT+*Bcbrn1-GFP*+*Bcrab7-RFP*	Expressing BcBRN1-GFP and BcRAB7-RFP in B05.10
WT+*Bcbrn1-GFP*+*Bcbrn2-RFP*	Expressing BcBRN1-GFP and BcBRN2-RFP in B05.10
WT+*Bcpks13-GFP*	Expressing BcPKS13-GFP in B05.10
WT+*Bcpks12-GFP*	Expressing BcPKS13-GFP in B05.10
WT+*Bcygh1-GFP*	Expressing BcPKS13-GFP in B05.10
WT+*Bcpks13-GFP*+*Bcrab5-RFP*	Expressing BcPKS13-GFP and BcRAB5-RFP in B05.10
WT+*Bcpks13-GFP*+*Bcpex3-RFP*	Expressing BcPKS13-GFP and BcPEX3-RFP in B05.10
WT+*Bcpks12-GFP*+*Bcpex3-RFP*	Expressing BcPKS12-GFP and BcPEX3-RFP in B05.10
WT+*Bcygh1-GFP*+*Bcpex3-RFP*	Expressing BcYGH1-GFP and BcPEX3-RFP in B05.10

### Assay of spore and sclerotium development.

The *B. cinerea* strains were cultured on CM at 23°C. Mycelial plugs (mycelia on plate agar) were excised from the edge of their 2-day-old colonies by a 5-mm-diameter cork borer, placed on fresh CM, and further cultured under light and dark conditions to collect conidia and sclerotia, respectively. The numbers of conidia and sclerotia formed were calculated 10 days after incubation. Conidia were collected by flooding mycelial colonies with water containing 0.1% Tween 40, filtering the suspension through 4 layers of sterile medical gauze, and then centrifuging the filtrate at 12,000 rpm for 5 min. The precipitated conidia were resuspended with sterilized water, and the number of conidia was counted using a hemocytometer.

### Pathogenicity assay.

Minimum Gamborg B5 (GB5; Coolaber, China) medium (0.6 g liter^−1^ GB5 salts, 10 mM glucose [pH 7.0]) was used for preparing spore suspensions for artificial inoculation in pathogenicity assays (52). Ten-microliter spore suspensions (10^6^ spores/ml) of the tested strains were placed on detached tomato leaves (Solanum lycopersicum) and incubated in a damp chamber at 23°C. Thirty leaves were used for inoculation with each fungal strain, and disease development was photorecorded at 2 or 4 days postinoculation (dpi). The decay area was measured by using ImageJ software (National Institutes of Health, USA).

### Assay of sclerotium germination.

Mycelial plugs of the test strains were cultured on CM at 23°C for 21 days in the dark. Mature sclerotia were collected with sterile tweezers and rinsed with sterilized water. The collected sclerotia of each strain were submerged in sterilized water in a six-well cell culture plate, followed by incubation at 23°C under continuous blacklight (UV-A, Phi-7383; Philips) for 21 days. The germination of sclerotia was monitored under a stereomicroscope and recorded by photography. The conidia generated from 30 sclerotia for each treatment were individually collected with distilled water containing 0.1% (vol/vol) Tween 40 and counted using a hemocytometer. Each experiment was repeated three times.

### Extraction and identification of melanin intermediates secreted in the medium.

Fungal cultures were extracted using a method described previously (53). Mycelial plugs of each strain were cultured in liquid YSS medium for 15 days, and the collected hyphae were lyophilized and powdered by vigorous shaking in a homogenizer with glass beads. The powders were immersed in a 0.2 M NaOH solution, and subsequently, an equal volume of acetone was added and mixed evenly. After 24 h, acetone was eliminated in a vacuum after acidification (pH 2) by HCl. The remaining solution was mixed with an equal volume of ethyl acetate for extraction. As for the culture filtrates, the solutions were adjusted to pH 2 by HCl, followed by extraction with an equal volume of ethyl acetate in a similar way. The extracts from either the mycelium biomass or the culture filtrate were dried and dissolved in ethyl acetate for TLC analysis, which was conducted with a mixture solution of ethyl ether-normal hexane-formic acid (60:40:1, by volume) as the mobile phase, followed by visualization under UV illumination or chromogenic reaction with 1% FeCl_3_. The target chemical was purified by column chromatography, and the structure and molecular weight were identified by nuclear magnetic resonance spectrometry (Bruker 500) and high-resolution mass spectrometry (Bruker MicroTOF-Q II LC MS) according to the instructions for the instruments.

### Detection of the subcellular localization of melanin synthesis enzymes.

Conidia of the test strains were suspended in GB5 liquid medium and adjusted to 10^5^ spores/ml. An aliquot (20 μl) of the spore suspension was dropped onto a glass slide and then placed in a moistened box for germination. Spore germination was observed by using a fluorescence microscope (Axio Imager Z2; Zeiss, Germany), using its light, fluorescence, and light/fluorescence-merged fields. Neutral-density filter set D/A (*d* = 25) was used during microscopic analysis. The excitation/emission wavelengths used were 488 nm/500 to 550 nm for GFP and 561 nm/570 to 620 nm for RFP. Moreover, the endosomes were stained with FM4-64 (Molecular Probes, AAT Bioquest, USA) and visualized under a microscope via excitation/emission wavelengths of 561 nm/570 to 620 nm according to a method described previously (54). In order to track vacuoles, the specific dye neutral red (CAS no. 553-24-2; Life Science Products & Services, China) was applied, and the stained samples were analyzed under a light microscope (55).

### Statistical analysis.

Statistical data are expressed as means ± standard errors (SE) from three replicates. Results for the analytical determinations were subjected to analysis of variance (ANOVA). All analyses were performed with SPSS software package v.17.0 (SPSS Inc., Chicago, IL, USA), using Tukey’s honestly significant difference (HSD) test to examine if differences between groups of samples were significant at a *P* value of <0.05.

## References

[1] Dean R, Van Kan JA, Pretorius ZA, Hammond-Kosack KE, Di Pietro A, Spanu PD, Rudd JJ, Dickman M, Kahmann R, Ellis J, Foster GD. 2012. The top 10 fungal pathogens in molecular plant pathology. Mol Plant Pathol 13:414–430. doi:10.1111/j.1364-3703.2011.00783.x. 22471698 PMC6638784

[2] Fillinger S, Walker A. 2016. Chemical control and resistance management of *Botrytis* diseases, p 189–216. *In* Fillinger S, Elad Y (ed), *Botrytis*—the fungus, the pathogen and its management in agricultural systems. Springer International Publishing, Cham, Switzerland.

[3] Leroux P. 2007. Chemical control of *Botrytis* and its resistance to chemical fungicides, p 195–222. *In* Elad Y, Williamson B, Tudzynski P, Delen N (ed), *Botrytis*: biology, pathology and control. Springer Netherlands, Dordrecht, Netherlands.

[4] Petrasch S, Silva CJ, Mesquida-Pesci SD, Gallegos K, van den Abeele C, Papin V, Fernandez-Acero FJ, Knapp SJ, Blanco-Ulate B. 2019. Infection strategies deployed by *Botrytis cinerea*, *Fusarium acuminatum*, and *Rhizopus stolonifer* as a function of tomato fruit ripening stage. Front Plant Sci 10:223. doi:10.3389/fpls.2019.00223. 30881367 PMC6405687

[5] Nakajima M, Akutsu K. 2014. Virulence factors of *Botrytis cinerea*. J Gen Plant Pathol 80:15–23. doi:10.1007/s10327-013-0492-0.

[6] Choquer M, Fournier E, Kunz C, Levis C, Pradier JM, Simon A, Viaud M. 2007. *Botrytis cinerea* virulence factors: new insights into a necrotrophic and polyphageous pathogen. FEMS Microbiol Lett 277:1–10. doi:10.1111/j.1574-6968.2007.00930.x. 17986079

[7] Veloso J, van Kan J. 2018. Many shades of grey in *Botrytis*-host plant interactions. Trends Plant Sci 23:613–622. doi:10.1016/j.tplants.2018.03.016. 29724660

[8] Amselem J, Cuomo CA, van Kan JA, Viaud M, Benito EP, Couloux A, Coutinho PM, de Vries RP, Dyer PS, Fillinger S, Fournier E, Gout L, Hahn M, Kohn L, Lapalu N, Plummer KM, Pradier JM, Quévillon E, Sharon A, Simon A, ten Have A, Tudzynski B, Tudzynski P, Wincker P, Andrew M, Anthouard V, Beever RE, Beffa R, Benoit I, Bouzid O, Brault B, Chen Z, Choquer M, Collémare J, Cotton P, Danchin EG, Da Silva C, Gautier A, Giraud C, Giraud T, Gonzalez C, Grossetete S, Güldener U, Henrissat B, Howlett BJ, Kodira C, Kretschmer M, Lappartient A, Leroch M, Levis C, et al. 2011. Genomic analysis of the necrotrophic fungal pathogens *Sclerotinia sclerotiorum* and *Botrytis cinerea*. PLoS Genet 7:e1002230. doi:10.1371/journal.pgen.1002230. 21876677 PMC3158057

[9] Elmer PAG, Michailides TJ. 2007. Epidemiology of *Botrytis cinerea* in orchard and vine crops, p 243–272. *In* Elad Y, Williamson B, Tudzynski P, Delen N (ed), *Botrytis*: biology, pathology and control. Springer Netherlands, Dordrecht, Netherlands.

[10] Holz G, Coertze S, Williamson B. 2007. The ecology of *Botrytis* on plant surfaces, p 9–27. *In* Elad Y, Williamson B, Tudzynski P, Delen N (ed), *Botrytis*: biology, pathology and control. Springer Netherlands, Dordrecht, Netherlands.

[11] Henson JM, Butler MJ, Day AW. 1999. The dark side of the mycelium: melanins of phytopathogenic fungi. Annu Rev Phytopathol 37:447–471. doi:10.1146/annurev.phyto.37.1.447. 11701831

[12] Eisenman HC, Casadevall A. 2012. Synthesis and assembly of fungal melanin. Appl Microbiol Biotechnol 93:931–940. doi:10.1007/s00253-011-3777-2. 22173481 PMC4318813

[13] Langfelder K, Streibel M, Jahn B, Haase G, Brakhage AA. 2003. Biosynthesis of fungal melanins and their importance for human pathogenic fungi. Fungal Genet Biol 38:143–158. doi:10.1016/s1087-1845(02)00526-1. 12620252

[14] Keller NP. 2019. Fungal secondary metabolism: regulation, function and drug discovery. Nat Rev Microbiol 17:167–180. doi:10.1038/s41579-018-0121-1. 30531948 PMC6381595

[15] Toledo AV, Franco MEE, Yanil Lopez SM, Troncozo MI, Saparrat MCN, Balatti PA. 2017. Melanins in fungi: types, localization and putative biological roles. Physiol Mol Plant Pathol 99:2–6. doi:10.1016/j.pmpp.2017.04.004.

[16] Casadevall A, Cordero RJB, Bryan R, Nosanchuk J, Dadachova E. 2017. Melanin, radiation, and energy transduction in fungi. Microbiol Spectr 5:FUNK-0037-2016. doi:10.1128/microbiolspec.FUNK-0037-2016. PMC11687467 28256187

[17] Liu GY, Nizet V. 2009. Color me bad: microbial pigments as virulence factors. Trends Microbiol 17:406–413. doi:10.1016/j.tim.2009.06.006. 19726196 PMC2743764

[18] Nosanchuk JD, Casadevall A. 2003. The contribution of melanin to microbial pathogenesis. Cell Microbiol 5:203–223. doi:10.1046/j.1462-5814.2003.00268.x. 12675679

[19] Jacobson ES. 2000. Pathogenic roles for fungal melanins. Clin Microbiol Rev 13:708–717. doi:10.1128/cmr.13.4.708-717.2000. 11023965 PMC88958

[20] Martin-Urdiroz M, Oses-Ruiz M, Ryder LS, Talbot NJ. 2016. Investigating the biology of plant infection by the rice blast fungus *Magnaporthe oryzae*. Fungal Genet Biol 90:61–68. doi:10.1016/j.fgb.2015.12.009. 26703899

[21] Ryder LS, Talbot NJ. 2015. Regulation of appressorium development in pathogenic fungi. Curr Opin Plant Biol 26:8–13. doi:10.1016/j.pbi.2015.05.013. 26043436 PMC4781897

[22] Chen Z, Nunes MA, Silva MC, Rodrigues CJ, Jr. 2004. Appressorium turgor pressure of *Colletotrichum kahawae* might have a role in coffee cuticle penetration. Mycologia 96:1199–1208. doi:10.1080/15572536.2005.11832868. 21148942

[23] Howard RJ, Valent B. 1996. Breaking and entering: host penetration by the fungal rice blast pathogen *Magnaporthe grisea*. Annu Rev Microbiol 50:491–512. doi:10.1146/annurev.micro.50.1.491. 8905089

[24] Mednick AJ, Nosanchuk JD, Casadevall A. 2005. Melanization of *Cryptococcus neoformans* affects lung inflammatory responses during cryptococcal infection. Infect Immun 73:2012–2019. doi:10.1128/IAI.73.4.2012-2019.2005. 15784542 PMC1087470

[25] Wang Y, Aisen P, Casadevall A. 1995. *Cryptococcus neoformans* melanin and virulence: mechanism of action. Infect Immun 63:3131–3136. doi:10.1128/IAI.63.8.3131-3136.1995. 7622240 PMC173427

[26] Heinekamp T, Thywißen A, Macheleidt J, Keller S, Valiante V, Brakhage AA. 2012. *Aspergillus fumigatus* melanins: interference with the host endocytosis pathway and impact on virulence. Front Microbiol 3:440. doi:10.3389/fmicb.2012.00440. 23346079 PMC3548413

[27] Pihet M, Vandeputte P, Tronchin G, Renier G, Saulnier P, Georgeault S, Mallet R, Chabasse D, Symoens F, Bouchara JP. 2009. Melanin is an essential component for the integrity of the cell wall of *Aspergillus fumigatus* conidia. BMC Microbiol 9:177. doi:10.1186/1471-2180-9-177. 19703288 PMC2740851

[28] Stappers MHT, Clark AE, Aimanianda V, Bidula S, Reid DM, Asamaphan P, Hardison SE, Dambuza IM, Valsecchi I, Kerscher B, Plato A, Wallace CA, Yuecel R, Hebecker B, da Glória Teixeira Sousa M, Cunha C, Liu Y, Feizi T, Brakhage AA, Kwon-Chung KJ, Gow NAR, Zanda M, Piras M, Zanato C, Jaeger M, Netea MG, van de Veerdonk FL, Lacerda JF, Campos A, Carvalho A, Willment JA, Latgé JP, Brown GD. 2018. Recognition of DHN-melanin by a C-type lectin receptor is required for immunity to *Aspergillus*. Nature 555:382–386. doi:10.1038/nature25974. 29489751 PMC5857201

[29] Schumacher J. 2016. DHN melanin biosynthesis in the plant pathogenic fungus *Botrytis cinerea* is based on two developmentally regulated key enzyme (PKS)-encoding genes. Mol Microbiol 99:729–748. doi:10.1111/mmi.13262. 26514268

[30] Zhou Y, Yang L, Wu M, Chen W, Li G, Zhang J. 2017. A single-nucleotide deletion in the transcription factor gene *bcsmr1* causes sclerotial-melanogenesis deficiency in *Botrytis cinerea*. Front Microbiol 8:2492. doi:10.3389/fmicb.2017.02492. 29312200 PMC5733056

[31] Zhou Y, Li N, Yang J, Yang L, Wu M, Chen W, Li G, Zhang J. 2018. Contrast between orange- and black-colored sclerotial isolates of *Botrytis cinerea*: melanogenesis and ecological fitness. Plant Dis 102:428–436. doi:10.1094/PDIS-11-16-1663-RE. 30673519

[32] Upadhyay S, Xu X, Lowry D, Jackson JC, Roberson RW, Lin X. 2016. Subcellular compartmentalization and trafficking of the biosynthetic machinery for fungal melanin. Cell Rep 14:2511–2518. doi:10.1016/j.celrep.2016.02.059. 26972005 PMC4805463

[33] Bartoszewska M, Opaliński L, Veenhuis M, van der Klei IJ. 2011. The significance of peroxisomes in secondary metabolite biosynthesis in filamentous fungi. Biotechnol Lett 33:1921–1931. doi:10.1007/s10529-011-0664-y. 21660569 PMC3173629

[34] Maggio-Hall LA, Wilson RA, Keller NP. 2005. Fundamental contribution of beta-oxidation to polyketide mycotoxin production in planta. Mol Plant Microbe Interact 18:783–793. doi:10.1094/MPMI-18-0783. 16134890

[35] Wang T, Ren D, Guo H, Chen X, Zhu P, Nie H, Xu L. 2020. CgSCD1 is essential for melanin biosynthesis and pathogenicity of *Colletotrichum gloeosporioides*. Pathogens 9:141. doi:10.3390/pathogens9020141. PMC7169410 32093195

[36] Evidente A, Bruno G, Andolfi A, Sparapano L. 2000. Two naphthalenone pentakides from liquid cultures of “*Phaeoacremonium aleophilum*”, a fungus associated with esca of grapevine. Phytopathol Mediterr 1:162–168.

[37] Jiao Y, Yoshihara T, Akimoto M, Ichihara A. 1994. Two phenolic compounds from *Valsa ambiens*. Biosci Biotechnol Biochem 4:784–785.

[38] Abou-Mansour A, Couché E, Tabacchi R. 2004. Do fungal naphthalenones have a role in the development of esca symptoms? Phytopathol Mediterr 1:75–82.

[39] Tokousbalides MC, Sisler HD. 1979. Site of inhibition by tricyclazole in the melanin biosynthetic pathway of *Verticillium dahliae*. Pestic Biochem Phys 11:64–73. doi:10.1016/0048-3575(79)90048-8.

[40] Kistler HC, Broz K. 2015. Cellular compartmentalization of secondary metabolism. Front Microbiol 6:68. doi:10.3389/fmicb.2015.00068. 25709603 PMC4321598

[41] Ramos-Pamplona M, Naqvi NI. 2006. Host invasion during rice-blast disease requires carnitine-dependent transport of peroxisomal acetyl-CoA. Mol Microbiol 61:61–75. doi:10.1111/j.1365-2958.2006.05194.x. 16824095

[42] Meijer WH, Gidijala L, Fekken S, Kiel JA, van den Berg MA, Lascaris R, Bovenberg RA, van der Klei IJ. 2010. Peroxisomes are required for efficient penicillin biosynthesis in *Penicillium chrysogenum*. Appl Environ Microbiol 76:5702–5709. doi:10.1128/AEM.02327-09. 20601503 PMC2935065

[43] Tang G, Chen Y, Xu JR, Kistler HC, Ma Z. 2018. The fungal myosin I is essential for *Fusarium* toxisome formation. PLoS Pathog 14:e1006827. doi:10.1371/journal.ppat.1006827. 29357387 PMC5794197

[44] Chen Y, Zheng S, Ju Z, Zhang C, Tang G, Wang J, Wen Z, Chen W, Ma Z. 2018. Contribution of peroxisomal docking machinery to mycotoxin biosynthesis, pathogenicity and pexophagy in the plant pathogenic fungus *Fusarium graminearum*. Environ Microbiol 20:3224–3245. doi:10.1111/1462-2920.14291. 29901274

[45] Tolleson WH. 2005. Human melanocyte biology, toxicology, and pathology. J Environ Sci Health C Environ Carcinog Ecotoxicol Rev 23:105–161. doi:10.1080/10590500500234970. 16291526

[46] Raposo G, Marks MS. 2002. The dark side of lysosome-related organelles: specialization of the endocytic pathway for melanosome biogenesis. Traffic 3:237–248. doi:10.1034/j.1600-0854.2002.030401.x. 11929605

[47] Ao J, Bandyopadhyay S, Free SJ. 2019. Characterization of the Neurospora crassa DHN melanin biosynthetic pathway in developing ascospores and peridium cells. Fungal Biol 123:1–9. doi:10.1016/j.funbio.2018.10.005. 30654952

[48] Salogiannis J, Egan MJ, Reck-Peterson SL. 2016. Peroxisomes move by hitchhiking on early endosomes using the novel linker protein PxdA. J Cell Biol 212:289–296. doi:10.1083/jcb.201512020. 26811422 PMC4748578

[49] Liu W, Soulie MC, Perrino C, Fillinger S. 2011. The osmosensing signal transduction pathway from *Botrytis cinerea* regulates cell wall integrity and MAP kinase pathways control melanin biosynthesis with influence of light. Fungal Genet Biol 48:377–387. doi:10.1016/j.fgb.2010.12.004. 21176789

[50] Patel RM, Heneghan MN, van Kan JA, Bailey AM, Foster GD. 2008. The pOT and pLOB vector systems: improving ease of transgene expression in *Botrytis cinerea*. J Gen Appl Microbiol 54:367–376. doi:10.2323/jgam.54.367. 19164879

[51] Cohrs KC, Burbank J, Schumacher J. 2017. A new transformant selection system for the gray mold fungus *Botrytis cinerea* based on the expression of fenhexamid-insensitive ERG27 variants. Fungal Genet Biol 100:42–51. doi:10.1016/j.fgb.2017.02.001. 28188884

[52] Muller N, Leroch M, Schumacher J, Zimmer D, Konnel A, Klug K, Leisen T, Scheuring D, Sommer F, Mühlhaus T, Schroda M, Hahn M. 2018. Investigations on VELVET regulatory mutants confirm the role of host tissue acidification and secretion of proteins in the pathogenesis of *Botrytis cinerea*. New Phytol 219:1062–1074. doi:10.1111/nph.15221. 29790574

[53] Kogej T, Wheeler MH, Lanisnik RT, Gunde-Cimerman N. 2004. Evidence for 1,8-dihydroxynaphthalene melanin in three halophilic black yeasts grown under saline and non-saline conditions. FEMS Microbiol Lett 232:203–209. doi:10.1016/S0378-1097(04)00073-4. 15033240

[54] Zheng W, Lin Y, Fang W, Zhao X, Lou Y, Wang G, Zheng H, Liang Q, Abubakar YS, Olsson S, Zhou J, Wang Z. 2018. The endosomal recycling of FgSnc1 by FgSnx41-FgSnx4 heterodimer is essential for polarized growth and pathogenicity in *Fusarium graminearum*. New Phytol 219:654–671. doi:10.1111/nph.15178. 29676464

[55] Weber RWS, Wakley GE, Pitt D. 1999. Histochemical and ultrastructural characterization of vacuoles and spherosomes as components of the lytic system in hyphae of the fungus *Botrytis cinerea*. Histochem J 31:293–301. doi:10.1023/A:1003713901179. 10461864

