# Supplementary material for: Compartmentalization of Melanin Biosynthetic Enzymes Contributes to Self-Defense against Intermediate Compound Scytalone in Botrytis cinerea
Source: mBio. 2021 Mar 23;12(2):e00007-21. doi: 10.1128/mBio.00007-21 (PMC8092192; doi:10.1128/mBio.00007-21)
Supplement: TABLE S1 [file mBio.00007-21-st001.docx]

**Table S1. Primer pairs used for target amplification by PCR**

| Primer pair | Nucleotide sequence | Target of amplification |
| --- | --- | --- |
| P1/P2 | 5’-GAATGGTGCTGATGATGC-3’/5’-CCACAGCTGCAGTCTAGAGCTGGTGATGATGAGGTTGGTGA-3’ | 5’UTR of *bcscd1* |
| P3/P4 | 5’-TCACCAACCTCATCATCACCAGCTCTAGACTGCAGCTGTGG-3’/5’-GTTTATAGCTTCCCTTGAACGGGATCCGCTTAGACAA-3’ | *hyg* |
| P5/P6 | 5’-TTGTCTAAGCGGATCCCGTTCAAGGGAAGCTATAAAC-3’/5’-TTGAACAGTAAACGAGGG-3’ | 3’UTR of *bcscd1* |
| P7/P8 | 5’-GTTGCGATGCGATGAGACA-3’/5’-GCGGGTAGCACCGAGAAGA-3’ | *bcscd1* |
| P9/P10 | 5’-CGGCGTAGGGTTGTTCC-3’/5’-TGGCGACCTCGTATTGG-3’ | *hyg* |
| P10/P11 | 5’-TGGCGACCTCGTATTGG-3’/ 5’-GTGCTCTGGTGTTAAGGC-3’ | *bcscd1-hyg* |
| P9/P12 | 5’-CGGCGTAGGGTTGTTCC-3’/5’- GTTCTGGGGATGAGAGATG-3’ | *hyg-bcscd1* |
| P13/P14 | 5’-ATGATTACGCCGAATTCGAGCTCGAGTCTTGAATGGTGCTG-3’/5’-CCTCACCCTTGGAAACCATGTTTATAGCTTCCCTTGAA-3’ | *bcscd1* |
| P15/P16 | 5’-GTTTCCAAGGGTGAGG-3’/5’- TTTGTAAAGTTCATCCATTCCC-3’ | *gfp* |
| P17/P18 | 5’-CGTCTAGAGCCGCATTCCCGATTC-3’/ 5’-AACGACGGCCAGTGCCAAGCTTC-3’ | *nat* |
| P19/P20 | 5’-AAAATGCTCCTTCAATATCACTAGCAAGTACATAGGCTTCTC-3’/5’-CTTACCTCGCCCTTGCTTACCATGCGACAAGCACAACCCTCCTTCGAAG-3’ | *bcrab5* |
| P21/P22 | 5’-CCATTATCAAGGAGTTCATGAG-3’/5’- GACGTTATATGCACCTGGGAGC-3’ | *rfp* |
| P23/P24 | 5’-AATGCTGCGTATGGTGGATG-3’/ 5’-GCGGTCGAATGGGTTTATCG-3’ | *f.ferg27* |
| P25/26 | 5’-ATGATTACGCCGAATTCGAGCTCTTCTAGAAGACGAAAGCGC-3’/5’-CCTCACCCTTGGAAACCATCATCATAGCTGCACCATCGA-3’ | *bcbrn1* |
| P27/P28 | 5’-AAAATGCTCCTTCAATATCACTAGTATATTGTCCCAAGACACAGC-3’/5’-ACTTACCTCGCCCTTGCTTACCATGCGCTTTCCACCACCATTGAGTTG-3’ | *bcbrn2* |
| P29/P30 | 5’-AAAATGCTCCTTCAATATCACTAGCGATTGGAAGTGCGAAGAGG-3’/5’-CTTACCTCGCCCTTGCTTACCATGCGGCAGGCACAGCCATCTCTATCG-3’ | *bcrab7* |
| P31/P32 | 5’-ATGATTACGCCGAATTCGAGCTGCCGAGGTCAAAATGATG-3’/5’-CGGGATTACTTACCTCACCCTTGGAAACCATCCCGGGTTTCTCGACATAGTAAGAGCCTCCCT-3’ | *bcpks12* |
| P33/P34 | 5’-ATGATTACGCCGAATTCGAGCTTGAGAAATATTTGACACA-3’/5’-TACTTACCTCACCCTTGGAAACCATCCCGGGTTTCTCGACATTTTGAGAGCTTGCTTCATAAG-3’ | *bcpks13* |
| P35/P36 | 5’-ATGATTACGCCGAATTCGAGCTGTGTGTATACGTCCGCGAAGCTC-3’/5’-GGAAACCATCCCGGGTTTCTCGATTCCATCACACTCTCCATCCAC-3’ | *bcygh1* |
| P37/P38 | 5’-AAAATGCTCCTTCAATATCACTTGTCATCTTGTGGCGTC-3’/5’-CTTACCTCGCCCTTGCTTACCATGCGCAAAGATGTTACTTTGTCAGCG-3’ | *bcpex3* |
